# A molecule perturbation software library and its application to study the effects of molecular design constraints

**DOI:** 10.1186/s13321-023-00761-5

**Published:** 2023-09-26

**Authors:** Alan Kerstjens, Hans De Winter

**Affiliations:** Laboratory of Medicinal Chemistry, Department of Pharmaceutical Sciences, University of Antwerp, Universiteitslaan 1, 2610 Wilrijk, Belgium

**Keywords:** Molecular design, Software library, RDKit, De novo molecule generation, Constraints, Topological perturbations, Chemical space, Molecular fingerprints

## Abstract

Computational molecular design can yield chemically unreasonable compounds when performed carelessly. A popular strategy to mitigate this risk is mimicking reference chemistry. This is commonly achieved by restricting the way in which molecules are constructed or modified. While it is well established that such an approach helps in designing chemically appealing molecules, concerns about these restrictions impacting chemical space exploration negatively linger. In this work we present a software library for constrained graph-based molecule manipulation and showcase its functionality by developing a molecule generator. Said generator designs molecules mimicking reference chemical features of differing granularity. We find that restricting molecular construction lightly, beyond the usual positive effects on drug-likeness and synthesizability of designed molecules, provides guidance to optimization algorithms navigating chemical space. Nonetheless, restricting molecular construction excessively can indeed hinder effective chemical space exploration.

**Supplementary Information:**

The online version contains supplementary material available at 10.1186/s13321-023-00761-5.

## Introduction

Many cheminformatics problems can be framed as searches through chemical space to find molecules with desirable properties. Examples include de novo molecular design [1, 2] and chemical library design [3, 4]. A popular way of exploring chemical space involves iteratively designing molecules and assaying them for properties of interest with one or multiple fitness or objective functions. A search or optimization algorithm, guided by the aforementioned objective functions, decides the regions of chemical space that are probed next based on the outcomes of the previous assays. Chemical space can be thought of as a multidimensional similarity-based arrangement of molecules. A molecule corresponds to a point in chemical space, and similar molecules, according to some criterion, are close to each other in chemical space. Depending on the application, different similarity criteria may be used to define chemical space [5–7]. Of interest to us is a chemical space defined based on molecular graph similarity, where molecules with similar topologies are proximal. It is believed that molecules with similar structures possess similar properties [8, 9]. Indeed, this has become one of the cornerstone theorems of molecular design. Hence, it’s common for optimization algorithms to wish to explore regions of chemical space surrounding reference molecules with promising properties, with the hopes of finding even more appealing molecules in their neighborhood. The problem of sampling molecules from these regions then becomes that of generating molecules “neighboring” the reference molecule. Traditionally this has been done by applying small modifications or perturbations to the reference molecule [1].

**Fig. 1:**
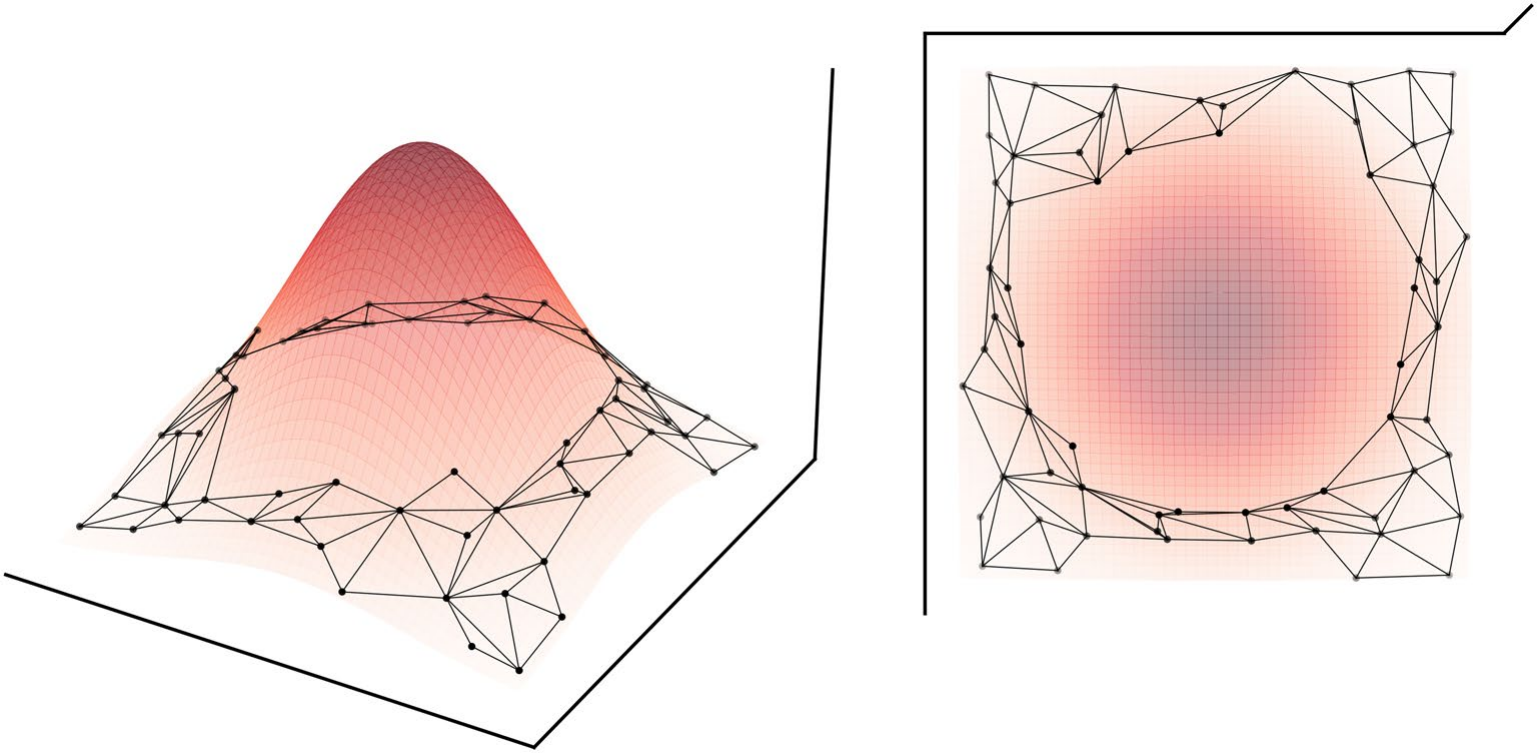
A section of graph-like chemical space with an excluded area (center). The exclusion stems from molecular construction constraints and corresponds to a maximum on an undesirability objective landscape (red)

A fitness landscape describes the fitness of all molecules in chemical space. The goal of drug discovery is to find molecules residing in the extrema of said landscape, that is, molecules for which the objective is either minimal or maximal. Drug discovery is inherently a multi-objective optimization problem, with the primary objective of designing biologically active molecules, and secondary objectives such as synthesizability and drug-likeness. Each of these objectives can be expressed explicitly through an individual fitness function, with a corresponding fitness landscape. One can optimize these objectives explicitly in parallel [10, 11]. However, defining and optimizing many objectives simultaneously can be challenging. Some authors try to evade the challenges of multi-objective optimization by considering explicitly only the primary objective and capturing the secondary objectives implicitly by constraining the molecular generation process to imitate known and desirable chemistry [12–22]. This effectively blocks access to certain areas of chemical space (Fig. 1). A large corpus of enumerated molecules with desirable secondary objectives exists [23–25], and it’s reasoned that constraining the molecular design process to only generate compounds similar to those in the corpus will yield molecules with desirable properties.

Many accounts describe the effectiveness of this approach to improve the drug-likeness and synthetic accessibility of generated molecules [12–22], but it’s not without drawbacks. The constraints imposed on molecule construction manifest themselves as barriers in search space, restricting the optimization algorithm’s freedom [22, 26]. These barriers may prevent accessing undesirable molecules, but inadvertently they may also hinder or impede discovering potentially appealing molecules, especially those that are most novel and resemble known chemistry the least.

**Fig. 2:**
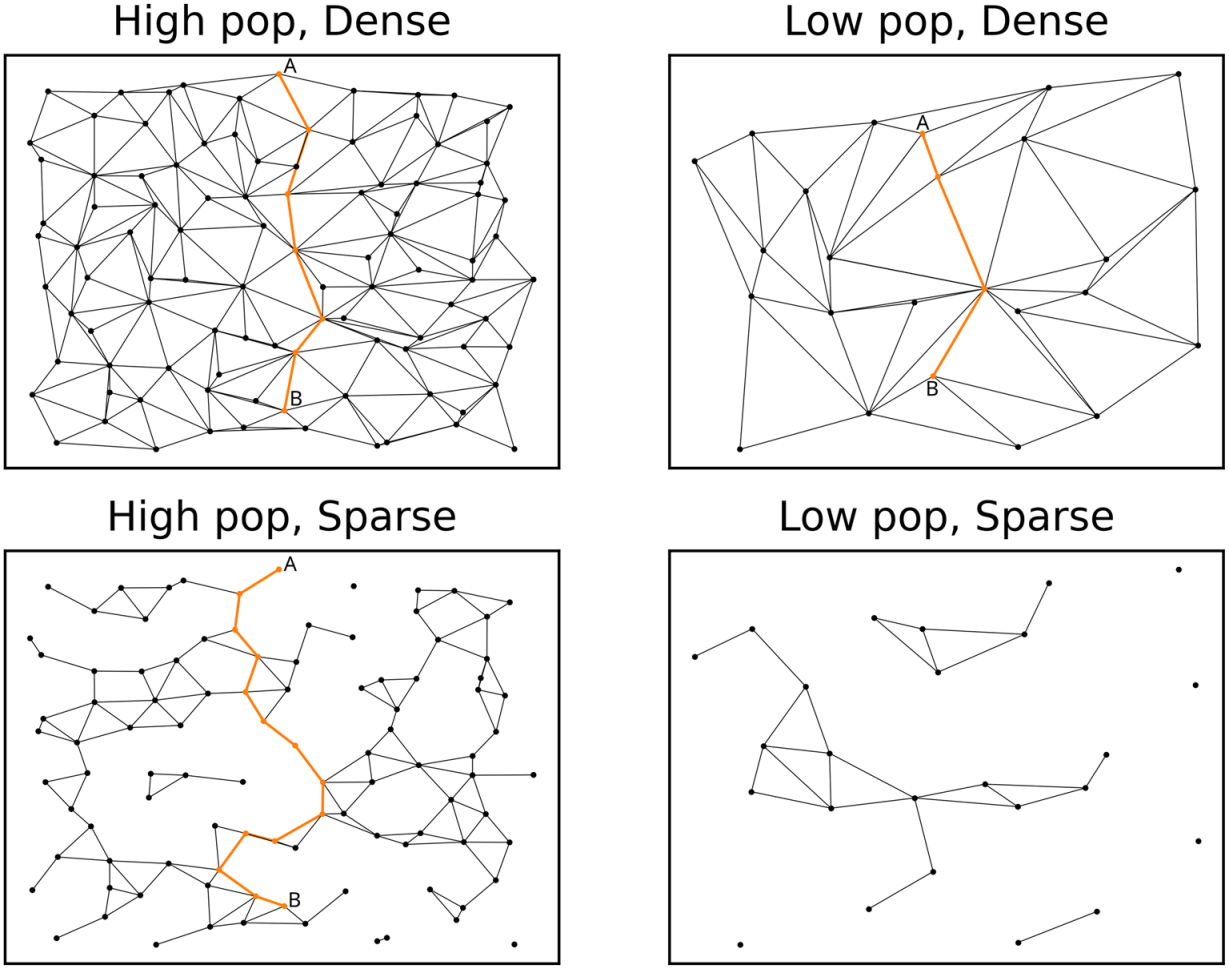
Examples of transition graphs of different population and density. The shortest path between two vertices **A** and **B** is highlighted in orange. Note that the path is shorter if the graph’s population is lower or the density is higher. As the population and density decrease the probability of two vertices being connected decreases

Consider some molecular generation scheme that can modify a reference molecule to yield related molecular entities. In this case chemical space can be visualized as a transition graph (previously termed a “morph graph” [27]), where vertices symbolize accessible molecules, and edges symbolize transitions between them (Fig. 2). The topology of this graph is dependent on the constraints of the chosen molecule generator. Generally speaking, atom-based approaches define a more populous graph than fragment-based approaches since a larger number of chemical states is accessible. The density of the graph (that is, the ratio between the existing number of edges and the theoretical maximum number of edges) depends on the strictness of the perturbation rules. Approaches with strict rules will define a sparse graph, while approaches with lax rules will define a dense graph.

Suppose that a chemical space search starts at a known molecule A. The goal is to find some unknown molecule B that exhibits good objective values. The more populous the transition graph, the more probable it will be that desirable molecules are part of it and therefore discoverable. The perfect optimization algorithm would define the shortest path between A and B. Such an ideal algorithm would benefit from a very populous and dense transition graph, as in these graphs paths between pairs of vertices tend to be shorter (Fig. 2).

Sadly, we don’t have access to these utopian search algorithms. In absence of omniscient oracles that reveal B and the path towards it, our algorithms must err on the side of exploration. Thorough exploration of very populous and dense graphs is computationally intractable. Trimming the size and density of the search graph in a chemically meaningful way could provide guidance to algorithms that otherwise would wander around unpromising regions of chemical space without a clear sense of direction.

In summary, when it comes to predicting the effect of molecular construction constraints on the fitness of the designed molecules, we are faced with two opposing hypotheses. The constraints may either hinder or facilitate chemical space exploration, and what the outcome will actually be is poorly understood.

Pieces of the answer lay scattered throughout the literature. Unfortunately, every study performs different experiments using different software, making it impossible to isolate the effect of any one variable. Attempts have been made to standardize experiments with benchmark suites [28–30], yet software is rarely standardized. Fully standardizing software is an impossible and arguably undesirable task as scientific methodologies are ever evolving. However, when it comes to graph-based molecule edition many commonalities can be found between different implementations.

We set out to create a software library for graph-based molecular edition providing the common denominator of functionality of previous implementations [3, 27, 31, 32]. We’ve named this library Molpert. Key considerations during the design were flexibility, extendibility, interoperability and ease of use. Molecule perturbations are atom-based, as fragment-based edition can be described in function of the former but not vice versa. Molecules are treated as graphs and modified without any sort of chemical considerations. This is by design as we didn’t want to impose our own biases and ways on others. Instead, users can specify themselves the properties the designed molecules ought to fulfill through means of arbitrary constraints. Mechanisms are foreseen to extend the functionality of the library should the provided functionality not suffice. Molpert is built on top of the RDKit, a highly popular and open-source cheminformatics toolkit [33], and integrates well with RDKit molecules. It has no other dependencies. A C + + and Python Application Programming Interface (API) are both provided.

In this paper we describe Molpert and showcase how it can be applied to cheminformatics research. Specifically, we use it to build an evolutionary algorithm for molecule design and try to answer the question: “What are the consequences of constraining atom-based molecular construction?”.

## Methods

### Property perturbations

Perturbations included in the library can be broadly classified into those changing the molecular graph’s annotations and those changing the graph’s topology. The former are trivial to understand and implement: each vertex (i.e. atom) and edge (i.e. bond) have a set of mutable numeric properties that are independent from the rest of the graph and can be freely changed. For atoms these properties are (1) the atomic number, (2) formal charge and (3) number of explicit hydrogens. For bonds the only property of interest is the bond type, which in most instances is equivalent to the bond order. Each property has a list of allowed values and associated sampling weights, both being user specified. By default the sampling weights are proportional to the property values’ frequencies in ChEMBL31 [23]. All properties have a corresponding perturbation to modify it.

Modifying the number of explicit hydrogens may seem superfluous as hydrogens are often treated implicitly. However, explicit hydrogens can be of importance to adjust the perception of implicit hydrogens. They are also one of the invariants used in topological fingerprint calculation [34, 35]. Hence, being able to modify the number of explicit hydrogens is essential for good interplay with fingerprint-based scoring functions.

### Topological perturbations

Topological perturbations refer to insertions and deletions of atoms and bonds. These operations could be performed by simply creating or destroying a single atom or bond. However, the resulting transformations may not match a chemist’s expectations about what these perturbations should entail.

Consider a molecular graph *G*(*V*,*E*) with vertices or atoms *V* and edges or bonds *E*. Naive implementation of topological perturbations may result, among other things, in a disconnected graph, that is, a graph in which there is a pair of atoms *v* and *w* between which no path exists. This is commonly undesirable unless the disconnected fragments represent salts.

**Fig. 3:**
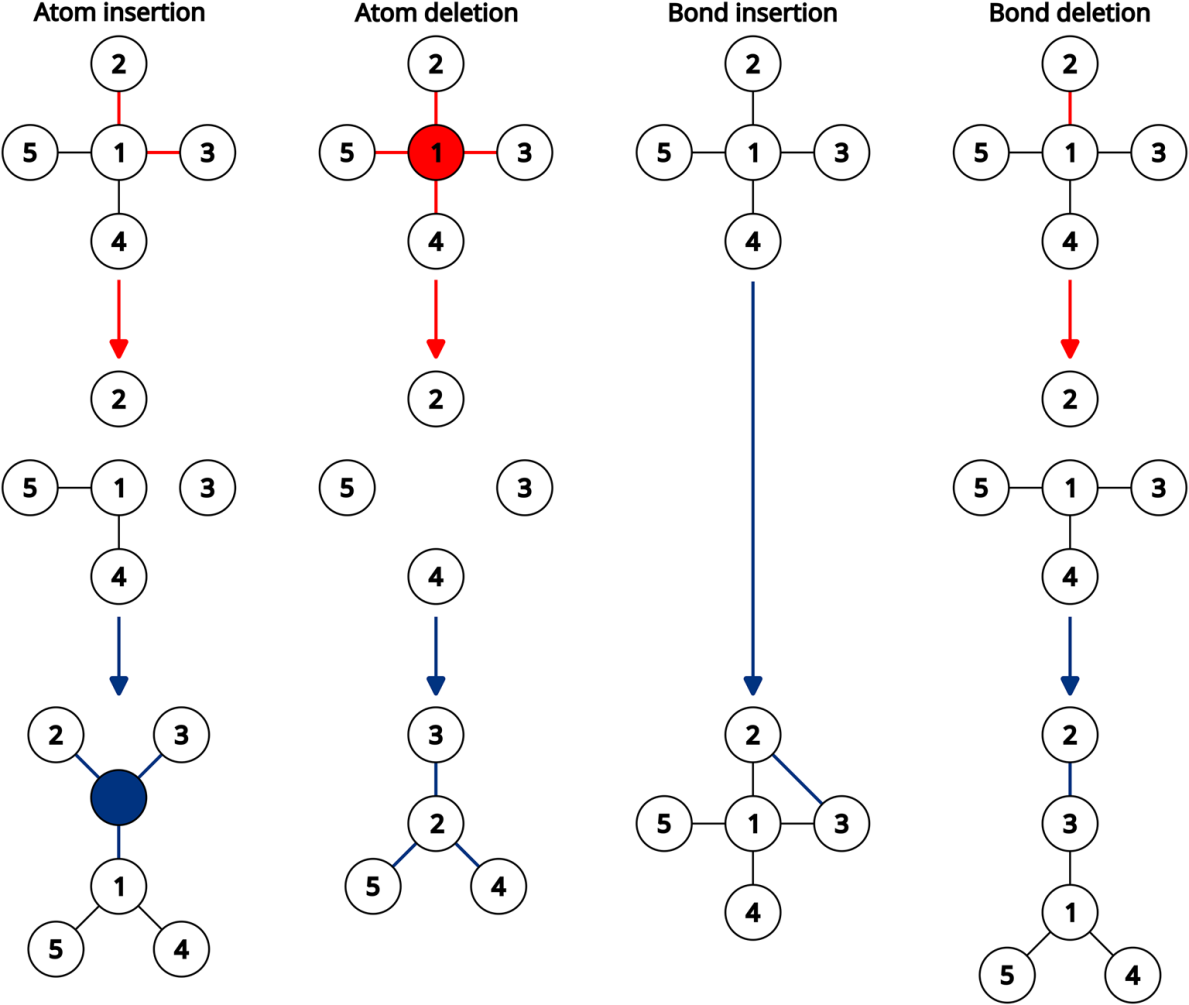
Examples of topological perturbations. Input and output molecules are depicted on the top and bottom respectively. Deleted atoms and bonds are highlighted in red while inserted atoms and bonds are highlighted in blue. In the atom insertion example N = {1, 2, 3} and p = {1}. In the atom deletion example N = {2, 3, 4, 5} and r = {2}

To ensure that the graph remains connected an atom insertion requires bond insertions as well. Hence, inserting a new atom *a* involves (1) selecting the atomic properties of *a*, (2) selecting a set of *k* existing atoms *N* to which *a* will bond with *k* new bonds *B* (*N ⊂ V*, |*N*|= *k, |B*|= *k*) and (3) selecting the bond types of *B*. Possible values for *a* and *B*’s properties are sampled from a list of allowed values. *k* ranges between 1 and a user specified parameter defaulting to 3 to avoid a combinatorial explosion in possible outcomes. Up to *k−1* cycles may be formed during this process. Cycle formation may be unwanted. For example, given an alkane one might want to extend the length of the chain without creating a cycle. In other words, one might want to insert an atom between other atoms. To do so we select as *N* a central atom *c* and some atoms *J* adjacent to *c* (*J* = *{j | c* ~ *j},* *N* = *c ∪ J*), and define a “dropped” atom *p* ∈ *N*. During insertion *a* and *B* are added and existing bonds between *p* and *N* *– p* are deleted. The destruction of some existing bonds allows the insertion of atoms in acyclic regions without the creation of cycles (Fig. 3). This only holds true if *N* is selected as described above such that all members of *N* are adjacent to *p* (*N* = *{n | n* ~ *p}*). If *N* comprises arbitrary atoms and two atoms *{v,w} ⊂ N* are separated by a topological distance *d(v,w)* ≥ *2* a cycle necessarily forms. Nonetheless, specifying a dropped atom can help in the design of more relaxed topologies that aren’t as densely packed with cycles.

Bond insertion is simple, as it only involves the selection of two atoms *v* and *w* where the topological distance between them *d(v,w)* > 1, the selection of a bond type and the creation of the bond. Once again, this necessarily creates a cycle of *d(v,w)* + *1* atoms (Fig. 3). A minimum and maximum *d(v,w)* may be specified. This provides the user with some control over the size of the resulting cycles but more importantly limits the number of possible outcomes.

Bonds are defined by a pair of atoms. Consequently, deleting one such atom *a* destroys the bond. Consider a set of atoms *N* adjacent to *a* (*N* = *{n | a* ~ *n}*). The degree *g* of *a* is defined as *g(a)* =|*N*|. If *g(a)* ≤ *1* it is peripheral, and if *g(a)* > *1* it is internal. Peripheral atoms and internal atoms that are members of a cycle can always be deleted without disconnecting the graph. Internal atoms that aren’t part of a cycle separate two parts of the graph through a unique path. Hence, their deletion would result in a graph disconnection. To prevent this the atom deletion may be followed by some bond formations. We define a “reconnection” atom *r* ∈ *V*, and create new bonds between *r* and *N* - *(N* ∩ *r)*. This ensures that a path passing through *r* exists between all pairs of atoms of *N* after the deletion of *a* and that the graph remains connected. Typically *r* ∈ *N*. Intuitively, this corresponds to deleting *a* and one of its neighbors *n*_*i*_* ∈ N* taking its place by bonding to the remainder of the neighbors *n*_*j*_* ∈ N* - *n*_*i*_ (Fig. 3). However, if the user allows it, one could also sample an arbitrary *r* within a given distance *d(a,r)* of *a*. This will result in the formation of a cycle of size *d(a,r)* when *d(a,r)* ≥ *3*.

Similarly to atom deletions, bond deletions result in graph disconnections if the bond isn’t a member of a cycle. To delete an acyclic bond without disconnecting the graph a new replacement bond *vw* must be formed. Similar operations have been previously described as “rerouting” the bond [27]. The newly bonded atoms* v* and *w* ought to be on opposite sides of the “chokepoint” defined by the deleted bond (Fig. 3). They must also be separated by a distance *d(v,w)* ≥ *2*, as otherwise the same bond would be recreated. The user can specify a maximum distance *d(v,w)* to alter the topology less drastically.

The described perturbations are sufficient to access the entirety of chemical space when executed in the right order. When sampled randomly specific long sequences of perturbations are statistically unlikely. It may be of interest to execute some of these sequences of perturbations as a unit. For example, one might want to insert a fragment corresponding to a specific functional group. It’s possible to combine the above elemental perturbations to create more complex operations.

### Molecule sanitization

Perturbations treat molecular graphs more like mathematical objects than chemical structures. Careless edition of the molecular graph is bound to result in chemically invalid structures. Notorious pain points include explicit hydrogen counts, stereochemistry and aromaticity. Cheminformatics toolkits like the RDKit store atom and bond properties as integers within atoms and bonds themselves. These properties may be sensible when first calculated, but can lose their meaning after modifying the molecular graph. We employ a post-perturbation sanitization procedure that either alters these properties to sensible values or deletes them altogether.

A heavy atom’s hydrogen count is modified to the value resulting in the lowest valid valence for said atom. The list of valid valences per element is provided by the RDKit. When no hydrogen count would result in a valid valence the count is set to zero. Chiral center stereochemistry labels are kept where possible. If a former chiral center is no longer chiral after a perturbation its stereochemistry label is erased. Newly formed chiral centers are not assigned any stereochemistry labels. Bond stereochemistry labels are always erased. Aromaticity presents the most egregious problem. Bonds may have been flagged as aromatic once upon a time, yet these flags are kept indefinitely even after modifying the molecule. The naive solution would be to convert aromatic bonds to single bonds once aromaticity has been broken. In the context of editing molecules, aromatic systems are fragile as most topological perturbations will cause aromaticity to be invalidated. On the other hand, creating an aromatic ring system is much more challenging, as it requires atoms and bonds of the right types to be placed in the right positions simultaneously. When modifying molecules stochastically the sequence of events leading to the creation of an aromatic ring system is highly unlikely. In practice this means that most designed ring systems won’t be aromatic, which is uncharacteristic of organic molecules.

**Fig. 4:**
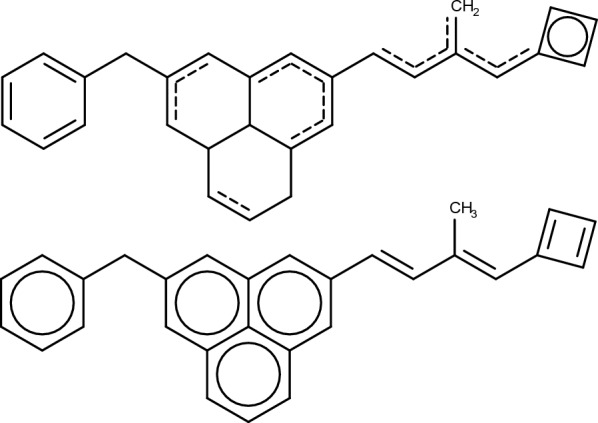
Aromaticity sanitization example. Aromatic bonds are depicted as dashed bonds. Aromatic ring systems where all bonds are aromatic are depicted with internal circles. Partially aromatic ring systems are either aromatized or kekulized depending on their “degree of aromaticity”. Bonds incorrectly labelled as aromatic are kekulized

Molpert handles aromaticity in two different ways, depending on the user’s preference. The simplest option is to work with kekulized molecules only, that is, molecules where aromatic systems are represented by alternating single and double bonds. Alternatively, one can work with “partially aromatic” molecules where the aromaticity flags are preserved, irrespective of whether they are valid at present time or not. For example, acyclic regions may be transiently labelled as aromatic. The former aromatic character of bonds is remembered and used to reestablish aromaticity in the future whenever conditions are right. When a molecule with valid aromaticity assignments is required, a sanitization procedure can be applied. Acyclic regions labelled as aromatic are kekulized. Rings are defined as components of the Smallest Set of Smallest Rings (SSSR) [36]. Rings that are correctly flagged as fully aromatic are left untouched. Kekulized rings fulfilling aromaticity criteria are aromatized. Partially aromatic rings are sorted in descending order according to their number of aromatic bonds and sanitized. If the number of aromatic bonds in the ring is greater than half and the ring otherwise meets the requirements to be aromatic it’s aromatized. Otherwise it is kekulized. Starting the sanitization process with the most aromatic rings allows aromaticity to propagate throughout fused ring systems (Fig. 4).

### Modes of operation

Perturbations are implemented as objects specifying how a molecule will be modified. These objects are callable and can be invoked when the perturbation ought to be executed. The user may construct these objects directly for fine-grained control over the outcome of a perturbation. For convenience we also provide factory functions that abstract away the details of constructing perturbations. Said factories can systematically enumerate all possible perturbations that could be applied to a molecule. Enumeration may be restricted to specific types of perturbations and/or atom/bond targets. When used deterministically all generated perturbations fulfilling the constraints are stored in a queue. When used stochastically the iteration order is randomized and the first generated perturbation fulfilling the constraints is returned. The randomization relies on a weighted shuffle in such a way that perturbations featuring common property values are most likely to be tried first [37]. This reduces the number of perturbations that need to be tried before one fulfilling the constraints is found.

### Constraints

While we designed the software to be able to generate any molecular graph, there may be instances where one wishes to use it to generate molecular graphs fulfilling specific criteria. This is enabled through constraints. In this context constraints are callback functions evaluating whether a molecule fulfills some arbitrary requirements. They take as input an atom, bond or molecule and return as output a boolean. A return value of “true” signals that the requirements are satisfied, whereas “false” signals the requirements aren’t met. Constraints may apply to one specific atom or bond. It’s therefore possible to constrain only certain parts of the molecule and to mix constraints as desired.

Constraints are enforced through trial and error. A queue of compatible perturbations is prepared. The perturbation at the front of the queue is applied to a copy of the molecule to simulate its outcome. The perturbed molecule is then forwarded to the constraints for evaluation. If any constraint evaluates to “false” the perturbed molecule is discarded and the next perturbation is simulated. This process repeats until a perturbation satisfying all constraints is found or the queue is empty. The stricter the constraints the higher the perturbation attrition rate and with it the computational performance degradation.

**Fig. 5:**
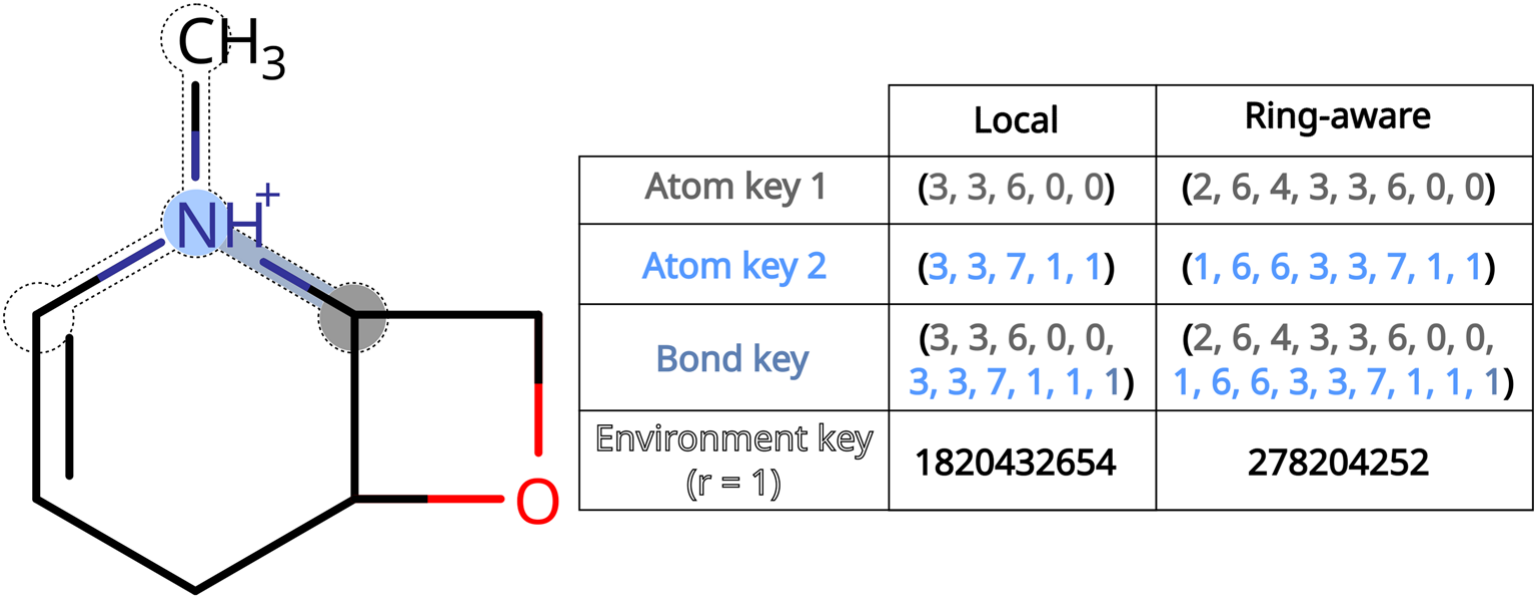
Example molecular keys. The color highlighted atoms are characterized with atom keys, and the color highlighted bond between them characterized with a bond key. The nitrogen’s circular atomic environment of radius 1 is shown as a dotted outline and characterized with the hash of its bonds’ keys, resulting in seemingly random numbers. For the meaning of each integer see Table 1

For our experiments we explored different variants of atom and bond constraints. The most basic constraints are valence constraints. Each element is assigned a maximum allowed valence, and any atoms of said element with a lower or equal valence are accepted, under the assumption that hydrogens can be added to pad the valence up to the closest valid value. The remainder of the constraints are key-based constraints. Atoms and bonds are characterized with atom and bond keys respectively (Table 1, Fig. 5). An atom key is a tuple of integer properties characterizing the atom. Depending on the properties used to define the key we distinguish between local atom keys made up of common atomic invariants [23, 24] (degree *D*, valence *V*, atomic number *Z*, formal charge *Q* and number of explicit hydrogens *H*) and ring**-**aware (RA) atom keys, which on top of the aforementioned atomic invariants include the number of SSSR rings the atom is involved (*R*) and the sizes of the smallest and largest SSSR rings it is involved in (*NR* and *XR* respectively). Bond keys are defined through combination of the bonded atoms’ keys and the bond’s type (*B*). Lastly, we also define atomic environment keys as the hashes of circular atomic environments, akin to the Extended Connectivity Fingerprint (ECFP) algorithm [35]. Environments of topological radii 1 (r = 1) and 2 (r = 2) were studied.

**Table 1:** Overview of the molecular keys used to characterize molecules and constrain molecular perturbation

Molecular key	Key structure
Local atom key	(D, V, Z, Q, H)
Ring-aware atom key	(R, XR, NR, D, V, Z, Q, H)
Local bond key (LB)	(D_1_, V_1_, Z_1_, Q_1_, H_1_, D_2_, V_2_, Z_2_, Q_2_, H_2_, B)
Ring-aware bond key (RAB)	(R_1_, XR_1_, NR_1_, D_1_, V_1_, Z_1_, Q_1_, H_1_, R_2_, XR_2_, NR_2_, D_2_, V_2_, Z_2_, Q_2_, H_2_, B)
Local environment key	Hash ({LB_1_, LB_2_ … LB_n_})
Ring-aware environment key	Hash ({RAB_1_, RAB_2_ … RAB_n_})

We determined the set of keys found in drug-like molecules, specifically ChEMBL31 [23], and recorded them in dictionaries, one for each key type. Given one such dictionary and a query molecule, a key based constraint calculates the same type of keys for the query molecule and compares said keys to the dictionary’s keys. If the query molecule exhibits keys that aren’t part of the dictionary the constraint evaluates to false and the query molecule is rejected.

### Property and perturbation sampling

Some molecular perturbations, namely property perturbations, atom insertions and bond insertions must sample atom and/or bond properties. The properties being sampled are atomic numbers, formal charges, explicit hydrogen counts and bond types. Property values are sampled from pre-defined sets of allowed values. When perturbations are enumerated deterministically each allowed value is used to construct one perturbation. When perturbations are generated stochastically a single allowed value is randomly sampled to construct a single perturbation, with each value having an associated sampling probability.

While the user may provide their own sampling values and probabilities, we provide some reasonable defaults. For each property we recorded the frequency of occurring values in ChEMBL31 [23], as well as the mode (i.e. the most frequent value). Property values occurring with a frequency larger than 0.01% are considered allowed and may be sampled with probabilities proportional to the values’ frequencies. The mode is taken as a default property value and, at the discretion of the user, may replace the list of allowed values to reduce the number of perturbations resulting from deterministic enumeration.

Property values for a specific atom or bond are sampled independently from the rest of the atom’s or bond’s properties and independently from their surrounding chemical environment. As an exception one may opt to sample atomic numbers and bond types with different probabilities depending on whether the atom/bond is part of a ring or not. The main motivation behind this exception is to preferentially place aromatic and double bonds in rings, and triple bonds in acyclic structures.

When generating perturbations stochastically the user may or may not specify the type of the perturbation. Should they choose to not do so the library will randomly sample a perturbation type for them. Property perturbations have sampling probabilities that are proportional to how often the property deviates from the mode. For example, since it’s rare to encounter charged atoms the probability of sampling a “formal charge change” perturbation is low. Conversely, since it’s relatively common to encounter non-carbon atoms the probability of sampling an “atomic number change” perturbation is comparatively high.

Weighted property sampling is supposed to reduce the probability of stochastically generating a constraint-infringing perturbation. To verify this assumption we took a subset of 10,000 ChEMBL molecules of varying sizes and generated 10 perturbations of each type for each molecule. We repeated the process twice sampling property values from either a uniform distribution or from the aforementioned ChEMBL-derived distribution. We then measured the perturbation rejection rate according to different molecular constraints.

### Chemical space connectivity

Stricter constraints are associated with sparser chemical spaces (Fig. 2). One can quantify the stringency of a set of constraints by calculating the average degree of the corresponding chemical transition graph. We stratified ChEMBL31 [23] according to the molecules’ heavy atom counts (HAC), and sampled 1000 random molecules every 5 HAC between 10 and 50 HAC. Two molecules are considered to be neighbors in chemical space if they are separated by a single edge in the chemical transition graph, that is, a single perturbation. Molpert was used to deterministically enumerate all perturbations applicable to each molecule of the aforementioned ChEMBL subsets. Said perturbations were subsequently executed to enumerate the molecule’s neighbors. Different perturbations may result in the same neighbor, but only unique neighbors were kept. The process was repeated using different sets of constraints, and the number of perturbations resulting in constraint-infringing neighbors was recorded. The average number of neighboring molecules, equal to the average degree of the transition graph, was taken as a measure of the constraints’ stringency.

### Benchmark

Two experiments were performed to evaluate the effects constraints have on atom-based chemical space exploration. To evaluate the effects constraints have on molecule fitness we developed an evolutionary algorithm using Molpert to mutate and recombine molecules. The algorithm was modelled after LEADD [22], a previously published evolutionary algorithm. Recombination isn’t a core part of Molpert, but simple digestion-based recombination operators [31, 38] are made available as addons. Every time a molecule is mutated we check if the resulting molecule satisfies the constraints. If it doesn’t the mutated molecule is discarded. The algorithm was tasked to design molecules maximizing the scores of GuacaMol goal-directed benchmark scoring functions [28]. Briefly, the GuacaMol goal-directed benchmark suite consists of 20 benchmarks with corresponding ligand-based objective functions that score molecules in the [0, 1] range, with higher scores being better. Some benchmarks demand the generation of a population of molecules, in which case the total benchmark score is calculated as a population weighted average. We opted out of this last step and took as score the fitness of the top molecule only. In our algorithm population diversity is enforced through means of a topological similarity threshold [22]. Due to this filter the remainder of the population is by design subpar and present solely to facilitate the evolution of the top molecule. Since evolutionary algorithms are stochastic one will presumably want to run multiple independent replicas anyways, sourcing the top molecule of each run. We ran the benchmark 50 times for each type of constraint recording the top molecule of each replica. Jobs were given a maximum of 72 h core hours. Some jobs for strict constraints failed to complete within this time, reducing the sample size (Additional file 1: Table S1). Differences in molecule fitness between the “no constraints” control group and constraints groups were analyzed using the non-parametric Kruskal–Wallis H-test [39] followed by pairwise Mann–Whitney U-tests [40] with Šidák correction [41]. α = 0.05 was taken as significance level and family-wise error rate.

To evaluate the chemical appeal and novelty of molecules we designed 1000 random molecules using each set of constraints. Said molecules, hereon forward referred to as Randomly Designed Molecules (RDM), were constructed through successive atom and bond insertions, aiming to create a molecule of 29 heavy atoms and 32 bonds, which corresponds to the average number of heavy atoms and bonds of molecules in ChEMBL31 [23]. Synthesizability and drug-likeness were assessed through means of the SAScore [42] and Quantitative Estimation of Drug-likeness (QED) [43] respectively. ChEMBL31 was used as reference synthesizable chemistry for SAScore calculations. Differences between distributions were analyzed with one-way Analysis Of Variance (ANOVA) [44] followed by Dunnett’s test [45]. α = 0.05 was taken as significance level and family-wise error rate. Chemical novelty was evaluated qualitatively by embedding the molecules into a 2D continuous chemical space and studying their location. Said chemical space was defined by characterizing molecules as binary 2048-bit ECFP4 fingerprints [35] and reducing their dimensionality with Principal Component Analysis (PCA) [46].

Optimizing molecules according to some objective function by design biases the regions of chemical space that are sampled. This is particularly true for the GuacaMol scoring functions, many of which incorporate topological similarity to some reference molecule as a component [22, 28]. We chose to study the chemical appeal and novelty of RDM as opposed to that of the optimized molecules resulting from the benchmark because we wanted to distinguish which effects are attributable to the constraints and which ones to the scoring function. Nonetheless, for completeness’ sake we repeated all analyses on the optimized molecules as well. Interested readers can find the corresponding results in the supplementary material (Additional file 1: Figures S2–S6, Tables S6–S7).

## Results

**Fig. 6:**
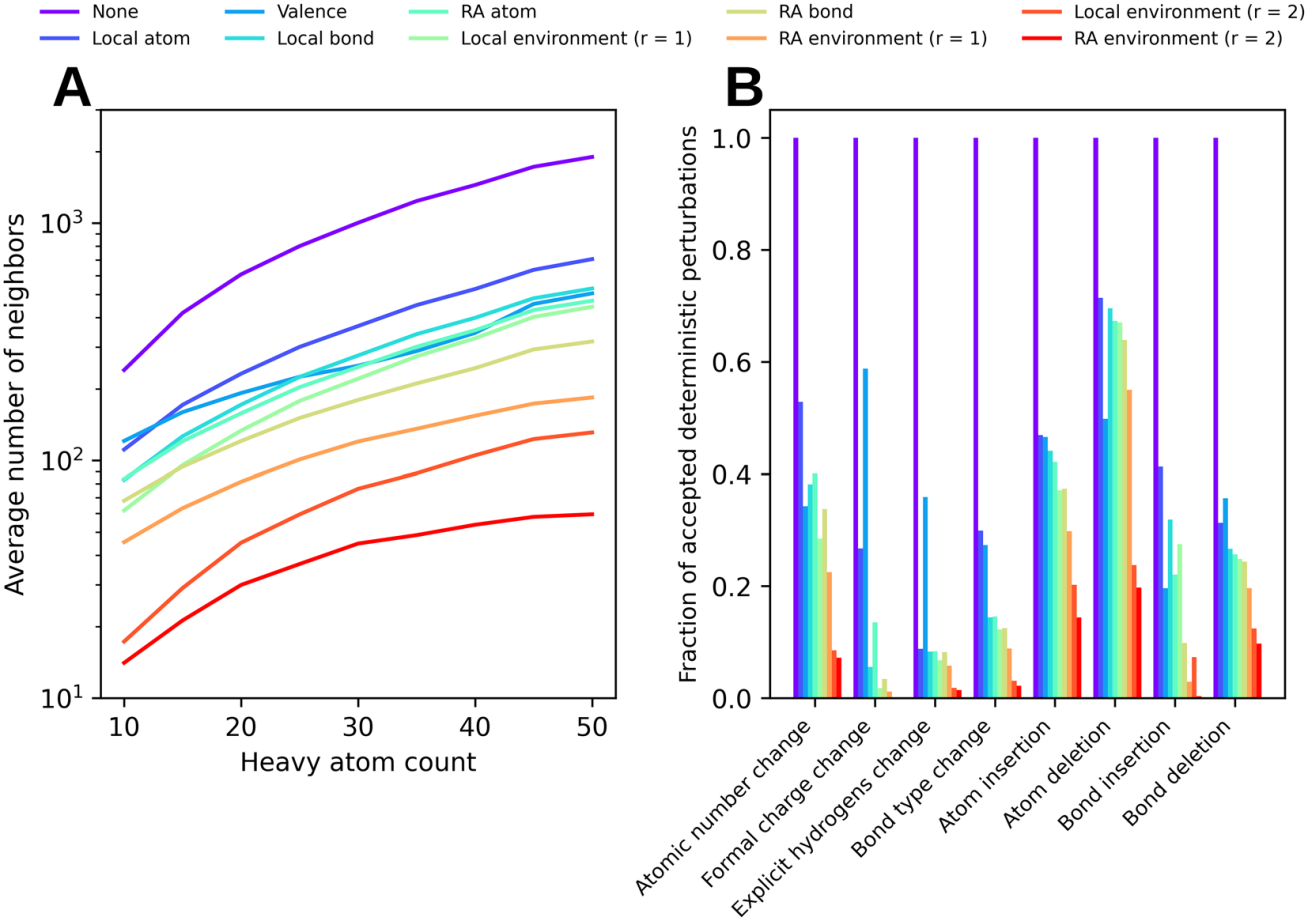
**A** Average number of neighboring molecules for molecules in ChEMBL based on their size and molecular constraints. The lower the number of neighbors the sparser the chemical transition graph. **B** Fraction of accepted perturbations broken down by perturbation type. The remainder of the perturbations were rejected by the molecular constraints

To rationalize the effects constraints have on molecular design it is important to study the extent to which they trim the chemical space transition graph. Figure 6A shows the average degree of said graph for different types of constraints. A higher average degree is indicative of a denser graph and therefore laxer constraints, whereas a lower average degree is indicative of sparser graphs and stricter constraints. The differences in graph density can be explained by discrepancies in the number of perturbations or moves that are rejected by the constraints Fig. 6B).

Generally speaking, constraints are stricter the more variables define the corresponding key. Accordingly, ring-aware (RA) constraints are stricter than local constraints, environment constraints are stricter than bond constraints, and bond constraints are stricter than atom constraints. Counterintuively valence constraints appear to be stricter than certain key-based constraints that encompass valence. This stems from differences in the definitions of valid valences. For valence constraints a rather conservative list of allowed valences hard coded within the RDKit is used. For key-based constraints allowed values are extracted from a large library of reference molecules. Should one find within this library a few examples of atoms with unusual valences this would suffice for said valences to be considered valid. Moving forward results will be color coded according to the constraint stringency order described in Fig. 6.

**Fig. 7:**
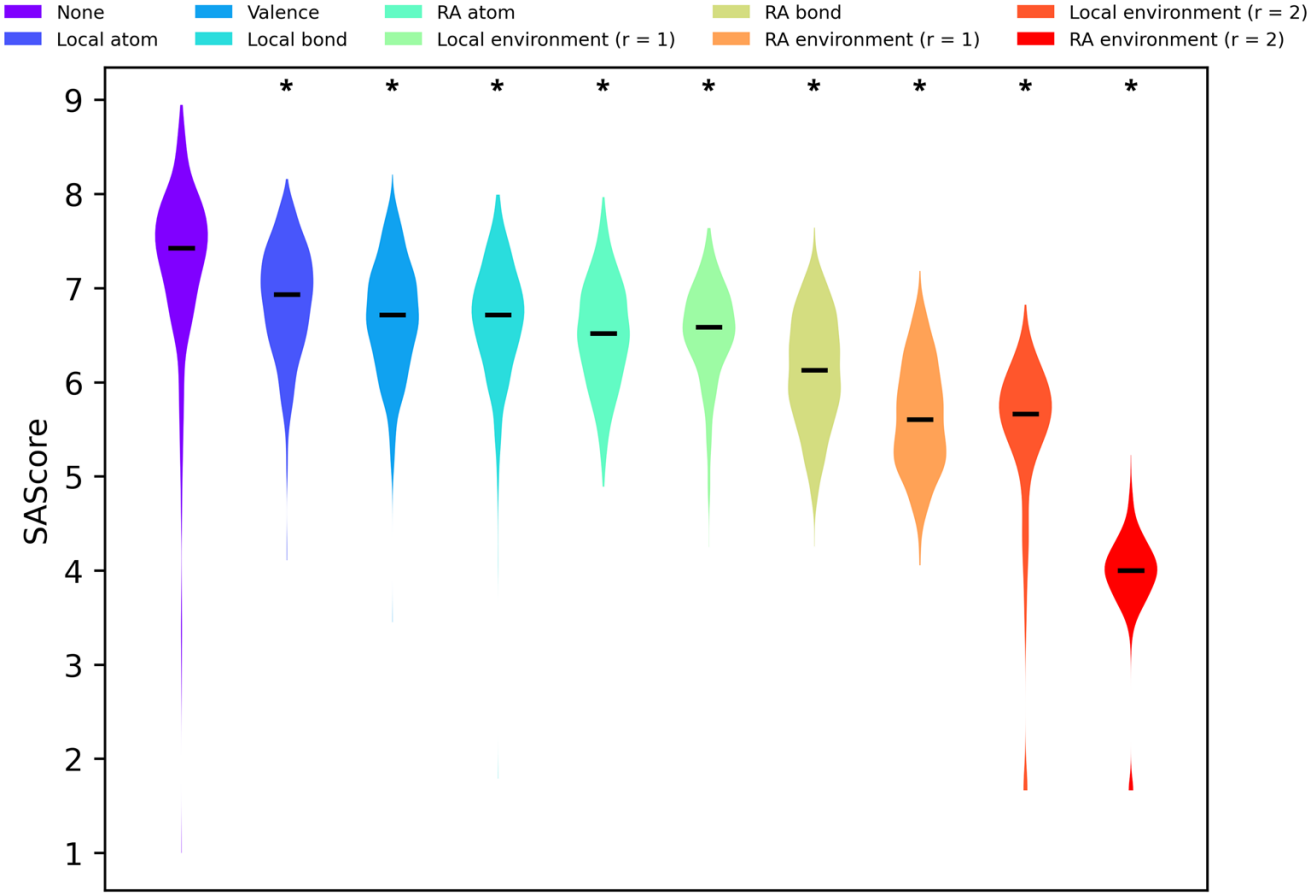
SAScore distributions of RDM using different types of constraints. Medians are shown as black lines. Lower SAScores are indicative of an easier synthesis. Stars on top of the distributions indicate statistically significant differences with the “no constraints” control group. A more detailed statistical analysis can be found in Additional file 1: Table S2

It is well established that constraining the way in which molecules are constructed increases the likelihood of designing chemically appealing molecules [12–22]. Figure 7 shows how the synthetic accessibility of RDM, as measured with the SAScore [42], increases with design constraints becoming stricter. Our constraints restrict the designed molecules to topological features present in reference molecules, with the differences between them being in the granularity of these features. As the granularity increases so does the algorithm’s ability to mimic the topology of reference molecules. Since the SAScore is partially calculated based on topological similarity to reference chemistry this result is to be expected.

**Fig. 8:**
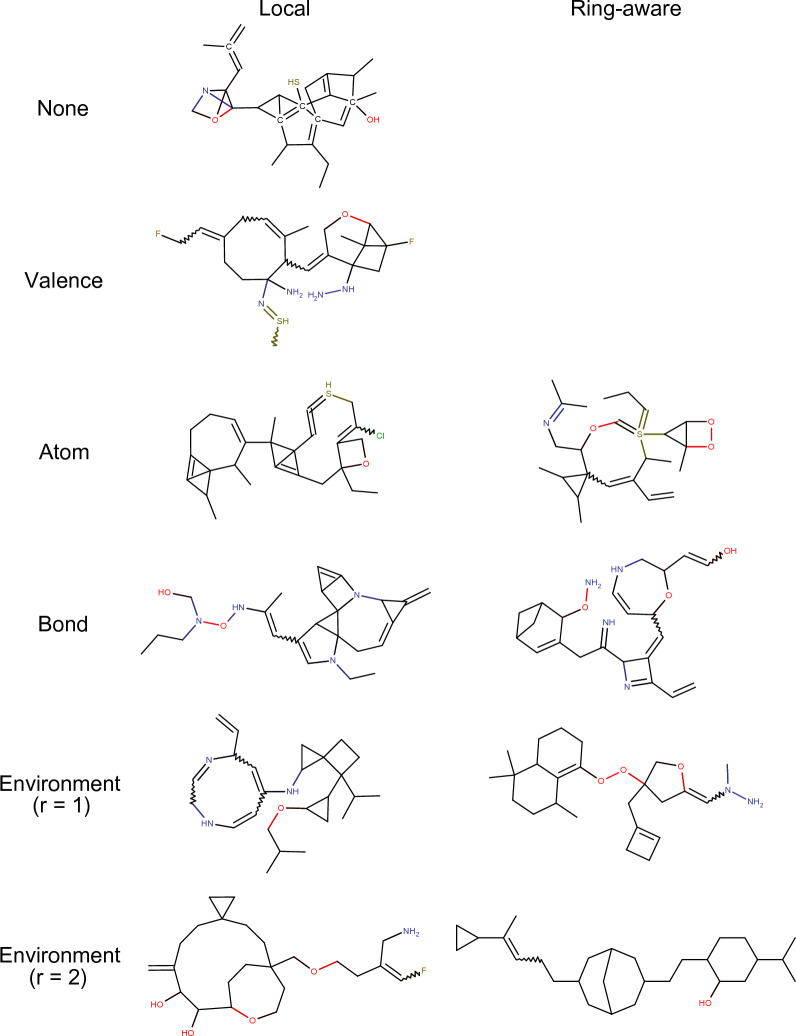
Examples of molecules designed by successive random atom and bond insertions using different types of constraints

Figure 8 shows some examples of molecules designed using different types of constraints. While subjective, molecules designed using stricter constraints are chemically more appealing. It has been noted that one of the main factors explaining chemists’ willingness to pursue synthesis or further development of a compound is the molecule’s ring complexity [47]. The use of ring-aware constraints discourages the design of complex ring systems. One could argue that the use of strict constraints leads to the design of “plain” molecules, rich in carbons and single bonds yet poor in functional groups. This foreshadows that excessively restricting molecular construction may be undesirable. Regardless of the constraints used, RDM are unlikely to contain aromatic systems (Fig. 8, Additional file 1: Table S4). As discussed previously, the creation of aromatic rings requires very specific arrangements of single and double bonds that are unlikely to occur by chance. This illustrates the value of the proposed partial aromaticity treatment.

**Fig. 9:**
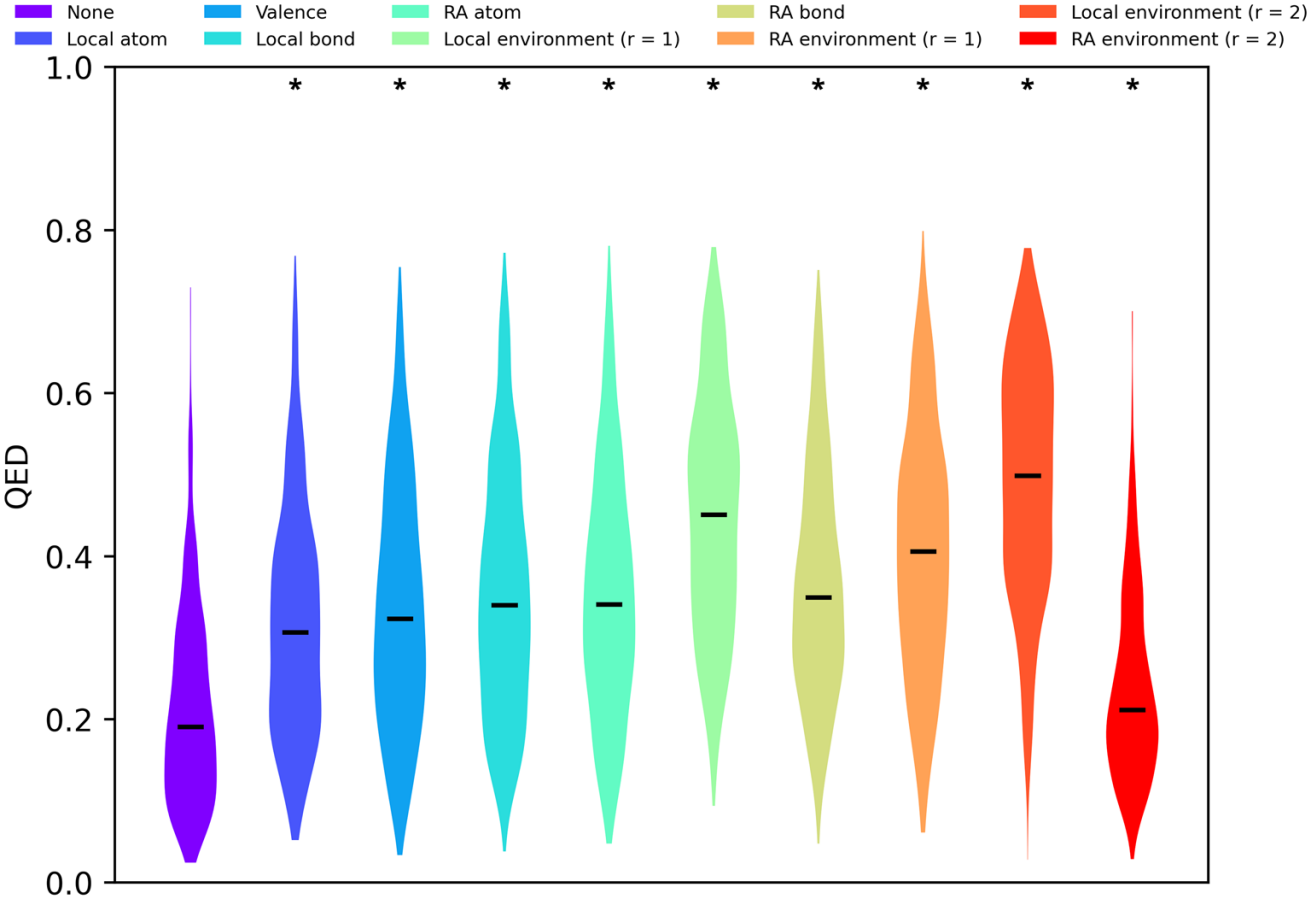
QED distributions of RDM using different types of constraints. Medians are shown as black lines. Higher values are indicative of more drug-like molecules. Stars on top of the distributions indicate statistically significant differences with the “no constraints” control group. A more detailed statistical analysis can be found in Additional file 1: Table S3

The use of constraints also seems to improve the drug-likeness of the designed molecules as measured with the QED [43] (Fig. 9). QED is calculated based mostly on physicochemical descriptors, yet our constraints don’t consider physicochemical descriptors explicitly. Further analysis reveals that the main driver for QED improvements is a reduction in the number of undesirable substructures (i.e. structural alerts) (Additional file 1: Table S4). A drop-off in QED is observed for RA environment (r = 2). This is explained by the designed molecules having over double the number of rotatable bonds one might expect to find in molecules designed with other constraints or drug-like molecules (Additional file 1: Table S4). RA environment (r = 2) constraints are so strict that oftentimes the only allowed atom insertion is that of carbons in existing hydrocarbon features, resulting in long and flexible molecules (Fig. 8).

We would like to clarify that within Molpert the only dependable source of molecule correctness are the molecular constraints. Weighted property sampling reduces the probability of stochastically generating perturbations that would infringe upon the constraints, but doesn’t prevent it (Additional file 1: Figure S7). Weighted sampling should thus be seen more as an algorithmic efficiency optimization than a strategy to design reasonable molecules.

**Fig. 10:**
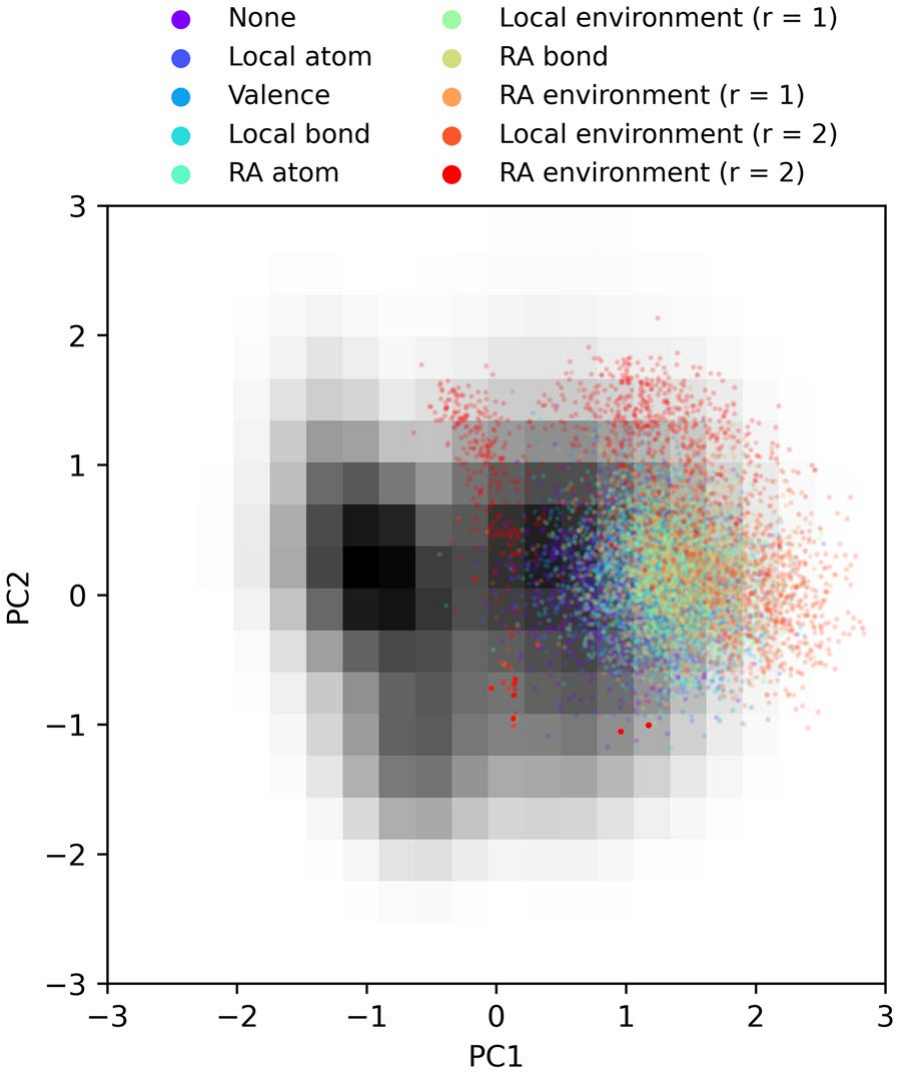
Positions of RDM in 2D PCA space. The grayscale grid represents the density of ChEMBL molecules in chemical space on a linear scale, with darker cells being more densely populated

One could be concerned that imitating reference chemistry stifles chemical innovation. To investigate this concern, we visualized the positions of designed molecules in a 2D chemical space, using ChEMBL [23] as a reference space (Fig. 10). There is some overlap between ChEMBL and designed molecules, but the latter are skewed towards the less densely populated areas of chemical space, regardless of the constraints used. It should be noted that a 2D projection of chemical space is overly simplistic, with distances between molecules appearing to be smaller than they truly are. Hence the designed molecules are more distinct from ChEMBL than what Fig. 10 might indicate. We believe that the designed molecules are sufficiently novel.

**Fig. 11:**
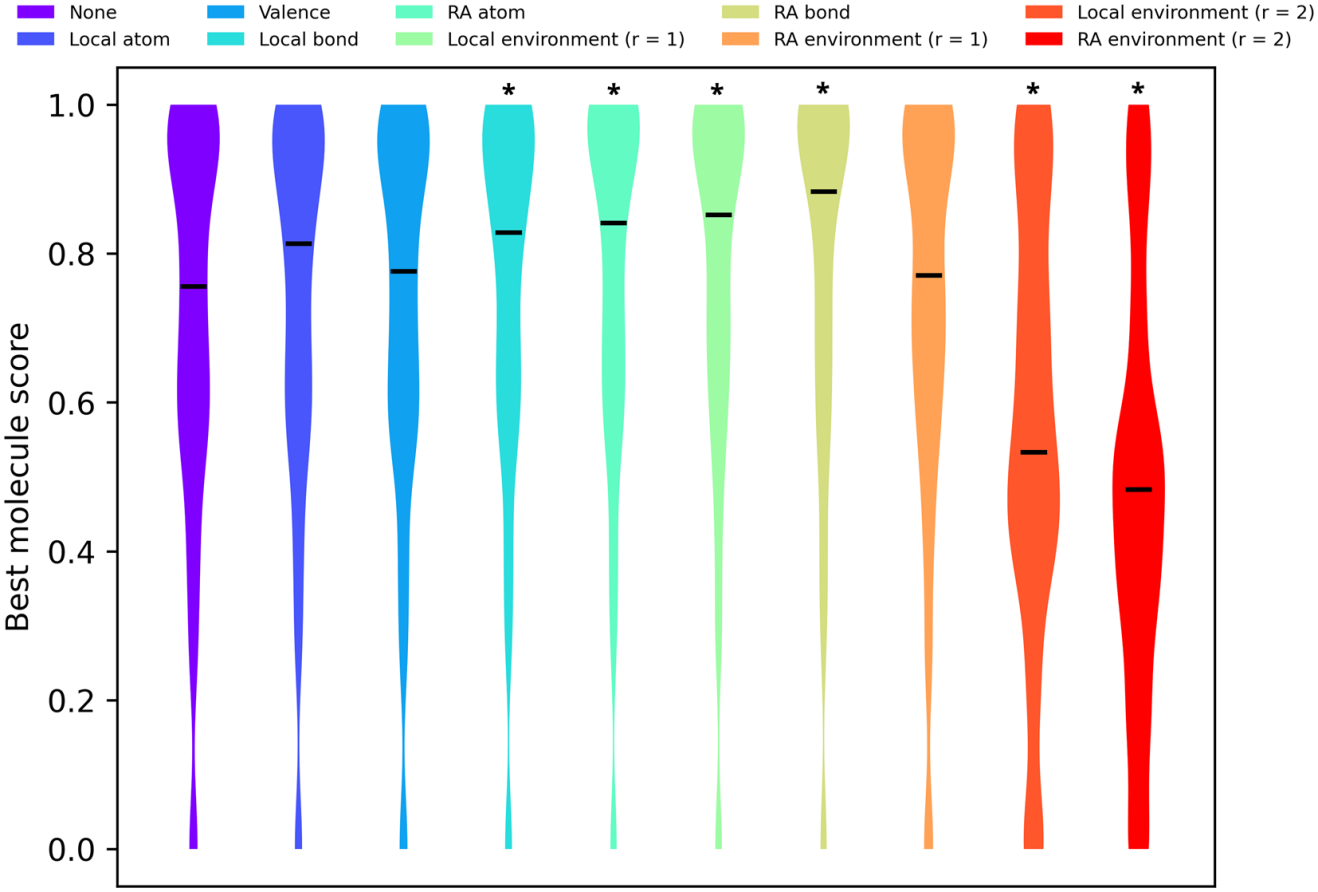
Distributions of top molecule scores, as assessed by the GuacaMol goal-directed scoring functions. Medians are shown as black lines. Only the best molecule of each population is included. The benchmark suite consists of 20 individual benchmarks, but for clarity’s sake the results of all benchmarks were aggregated. A per-benchmark breakdown can be found in Additional file 1: Figure S1. Stars on top of the distributions indicate statistically significant differences with the “no constraints” control group. A more detailed statistical analysis can be found in Additional file 1: Table S5

The effect constraints have on compound fitness during molecular optimization is poorly understood. Figure 11 shows the optimization power of a Molpert-based evolutionary algorithm in the GuacaMol benchmark suite [28] using different types of constraints. As a reminder, constraints are enforced every time a molecule is mutated. Using mild constraints, that is, anything between local and RA bond constraints, leads to significantly improved molecule fitness over unconstrained molecular design. RA bond constraints performed best, followed closely by local environment (r = 1) and RA atom constraints. RA environment (r = 1) constraints are equivalent to unconstrained molecular design in terms of molecule fitness. Stricter constraints, namely environment (r = 2) constraints are markedly worse.

The results in Fig. 11 suggest that there is a constraint stringency sweet spot that trims the search graph in just the right way to facilitate the optimization process. Upsettingly the exact location of this sweet spot depends on the individual benchmark (Additional file 1: Figure S1). The most common pattern is a fitness maximum at some constraint stringency middle point such as RA bond constraints, with laxer and stricter constraints both performing worse. Even in the cases where fitness is unaffected by constraint choice most constraints seem to be tolerated. This is an encouraging result as the primary use of constraints in molecular design is to increase the likelihood of designing drug-like and synthesizable molecules.

**Fig. 12:**
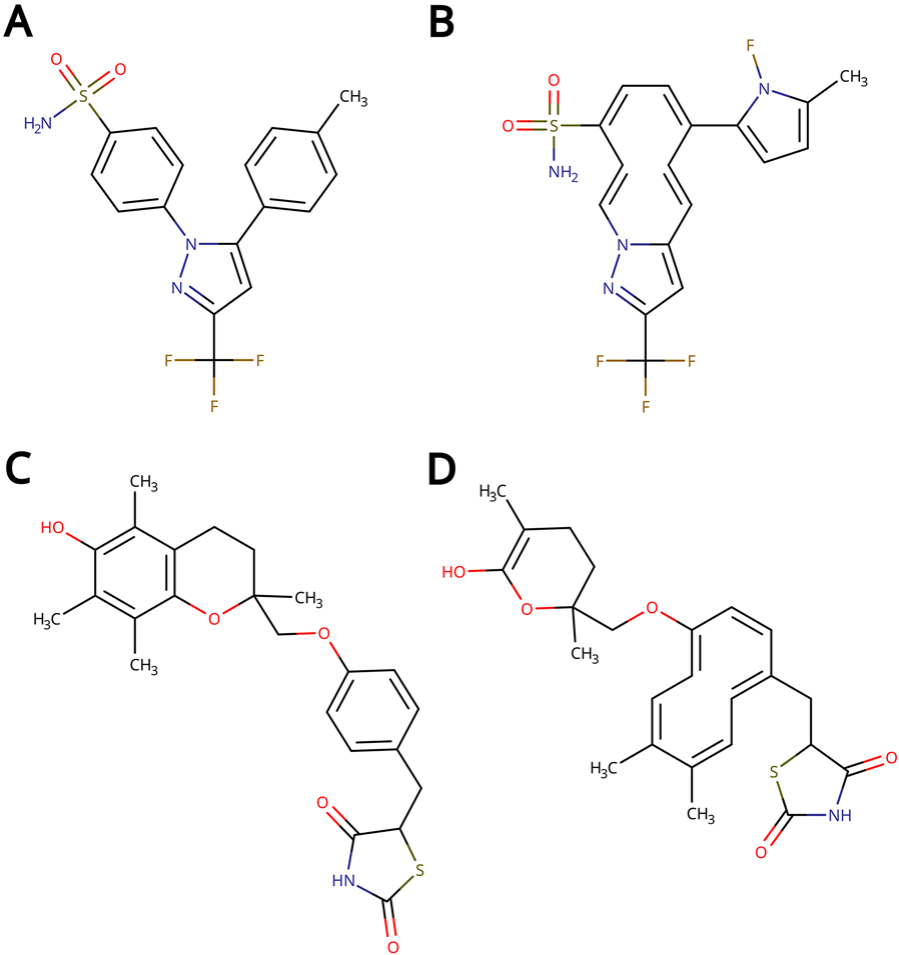
Celecoxib (**A**), troglitazone (**C**) and examples of molecules designed during their rediscovery benchmark using local bond constraints. The designed molecules (**B**) and (**D**) score relatively high (0.62 and 0.69 respectively) due to the presence of common chemical features albeit in different positions. Note that the 10-membered cycles in (**B**) and (**D**) are deemed aromatic by Hückel’s rule [48] and the RDKit, despite not being aromatic due to ring strain [49]

Two peculiar cases are those of Celecoxib and Troglitazone rediscovery, where very pronounced fitness differences are observed between local and RA constraints (Additional file 1: Figure S1). Visual inspection of the designed molecules reveals that when using local constraints the algorithm correctly rediscovers many of the reference molecule’s features, but proposes alternative ring systems. In rediscovery benchmarks the goal is to re-design a reference molecule, with the score being given by the topological similarity to the reference molecule. Topological similarity is assessed through means of ECFP4 fingerprints similarity [35], with two molecules being similar if they share many chemical features. Crucially, it is not required for the features to be in the same position for two molecules to be deemed similar. Celecoxib and troglitazone possess multiple benzene rings, with paths of aromatic carbons as features. The algorithm is rewarded for designing molecules with aromatic carbons, but this reward is the same regardless of the topology and size of the ring systems. Limiting the sizes of designed rings with RA constraints can prevent the algorithm from being led astray and towards macrocycles by the scoring function (Fig. 12).

**Fig. 13:**
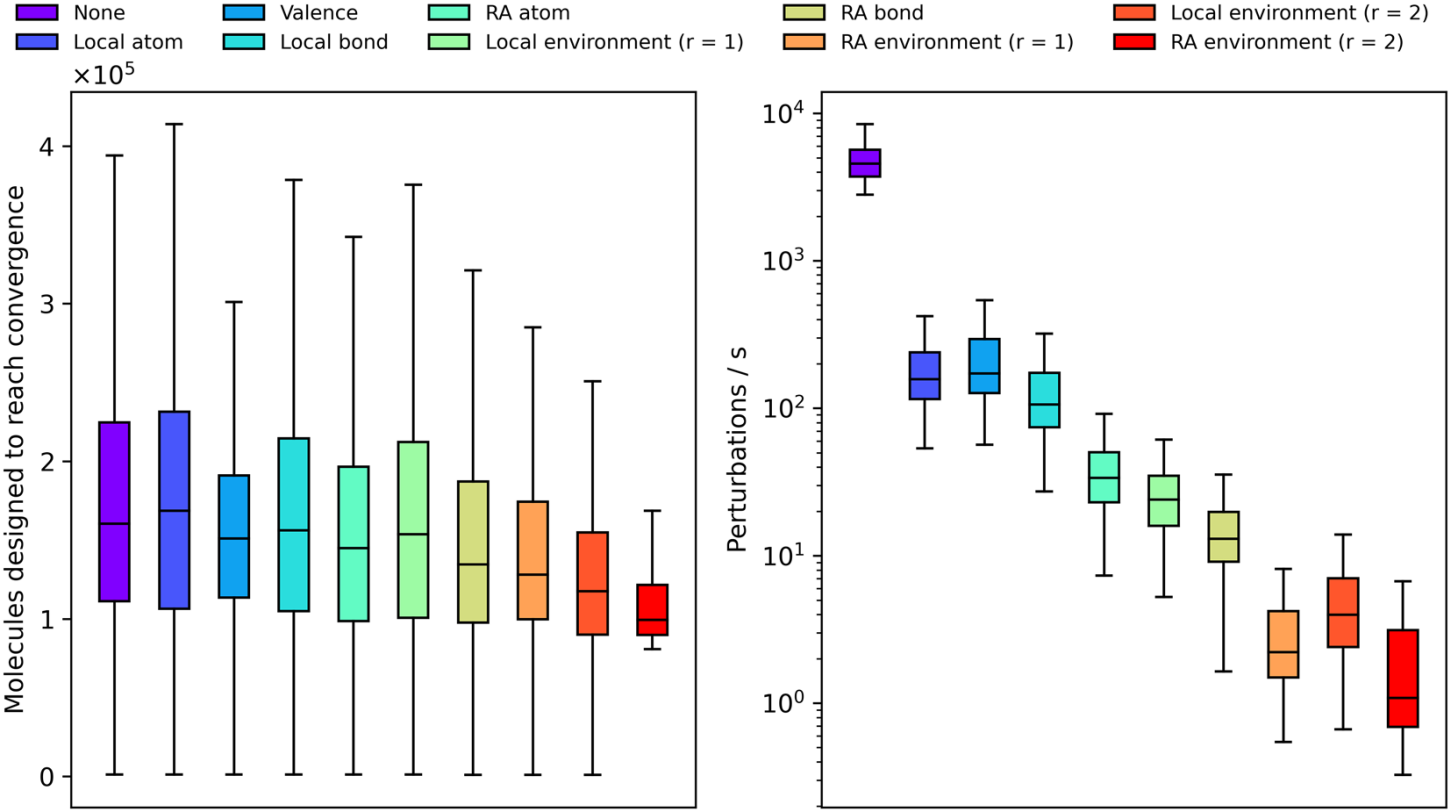
Number of molecules designed to reach convergence (left) and the number of perturbations executed per second (right) stratified per constraint type. Performance numbers are for a single-threaded workload on an AMD Epyc 7452 CPU clocked at 2.35 GHz

It’s worth noting that molecular design constraints can add considerable computational overhead (Fig. 13). This is especially true for Molpert since constraints are enforced in a naive fashion. The slow down stems from a higher perturbation rejection rate for stricter constraints, prolonging the search for a suitable perturbation. Interestingly the number of molecules designed before the algorithm reaches convergence is moderately lower for stricter constraints. For the vast majority of objective functions this decrease is insufficient to offset the increased perturbation cost. Nonetheless, when working with very expensive objective functions the cost of perturbing molecules can be negligible compared to the cost of scoring them, making the use of constraints as convergence acceleration strategy an appealing proposition.

## Discussion

Our results indicate that moderately constraining molecular construction has a net positive effect as it increases both the synthesizability and fitness of designed molecules. Nonetheless one must care to not choose excessively strict constraints as this can cause a sharp degradation of molecule fitness. As a guideline we recommend constraining bond or small environment properties and, if necessary, ring topologies. However, while the results presented herein apply to atom-based evolutionary algorithms, they may not be extrapolatable to alternative molecular optimization schemes. Evolutionary algorithms are powerful optimizers capable of navigating complex search spaces. Other algorithms such as tree searches may be less tolerant of barriers in search space and therefore construction constraints. We developed a simple tree search algorithm to test this hypothesis but found the fitness of the designed molecules too poor to extract any useful conclusions. Evolutionary algorithms are heuristic gradient-free optimization algorithms. They wander around chemical space until they stumble upon good solutions by chance. For an algorithm lacking a sense of direction the very dense chemical spaces characteristic of unconstrained molecular construction can seem like a maze with many “false paths”. Gradient-based optimization algorithms do have a sense of direction and may benefit from unconstrained molecular design.

Versatility was a major consideration when designing the Molpert. Unfortunately, in software development versatility often comes at the cost of computational efficiency. Molpert is efficient at unconstrained molecular design, but this efficiency decreases with constraint stringency. Despite the decreased efficiency molecular design remained a tractable task. If one were to settle on an immutable set of constraints that molecules must fulfill it would indubitably be possible to write more specialized and performant algorithms. Should one wish to do so we would recommend using Molpert to build a prototype and confirm the effect of the envisioned algorithm and/or constraints before committing resources to developing a performant solution. We wanted to be able to re-use the code base in projects with differing requirements. Anecdotally during the development of the software we went through multiple iterations of more efficient yet less flexible constraint implementations, but kept encountering use cases that couldn’t be covered by those alternative systems. This cemented our conviction to support truly arbitrary constraints.

In lieu of using constraints one could embrace unconstrained molecular design. When using stochastic molecule generators molecule fitness follows a distribution. While unconstrained molecular design may yield less fit molecules on average, it still may occasionally result in high scoring molecules (Fig. 11). Sampling more times from a distribution with a lower median may be a superior strategy to sampling fewer times from a distribution with a higher median, provided that the variance is large enough (Fig. 11). The faster unconstrained molecular generation allows one to roll the dice more often in the same amount of time. Biasing the design towards synthesizable molecules remains possible in absence of constraints by incorporating synthesizability into the objective function [50, 51]. Ideally the objective function should be able to evaluate the fitness of potentially invalid molecules resulting from unconstrained molecular design. Our benchmark shows that at least some scoring functions are able to do so, and we hypothesize that most ligand-based scoring functions will share this ability. Machine learning models may excel at this task given their interpolation capabilities. Structure-based scoring functions requiring conformation generation or relying on knowledge-based parameters, such as molecular mechanics, might be less suited for this purpose. Even then one could return null or negative fitness values when a molecule can’t be evaluated, in which case the objective function acts as a constraint itself. Lastly, while it’s usually undesirable to design difficult to synthesize or even chemically invalid molecules some readers may find use in expressly generating these sorts of unreasonable molecules, for example as negative training data for machine learning models [52].

## Conclusion

Imposing moderate constraints on molecule construction techniques has a net positive effect, as it improves both the fitness and chemical appeal of the constructed molecules. One must care to not use excessively strict constraints as doing so would have a negative effect on molecule fitness. We demonstrated how Molpert can be used to develop molecular design applications. We believe Molpert is a useful tool for cheminformatics software development, especially for prototyping, and hope it will relieve researchers from the burden of reinventing molecular graph edition.

### Supplementary Information

**Additional file 1:** Statistical test results, per-benchmark molecular fitness analysis, and synthesizability, drug-likeness and novelty analyses of benchmark-optimized molecules.

## Data Availability

Project name: Molpert, Project home page: https://github.com/AlanKerstjens/Molpert, Archived version: Molpert_0.0.1, Operating system(s): Platform independent, Programming language(s): C + + , Python, Other requirements: RDKit cheminformatics toolkit [33], License: AGPL 3.0

## Abbreviations

API: Application Programming Interface
SSSR: Smallest Set of Smallest Rings
RA: Ring-aware
ECFP: Extended Connectivity FingerPrint
HAC: Heavy atom count
RDM: Randomly designed molecules
QED: Quantitative estimation of drug-likeness
ANOVA: Analysis of Variance
PCA: Principal Component Analysis
