# Supplementary material for: A molecule perturbation software library and its application to study the effects of molecular design constraints
Source: J Cheminform. 2023 Sep 26;15:89. doi: 10.1186/s13321-023-00761-5 (PMC10523775; doi:10.1186/s13321-023-00761-5)
Supplement: Supplementary file 1 — Additional file 1: Statistical test results, per-benchmark molecular fitness analysis, and synthesizability, drug-likeness and novelty analyses of benchmark-optimized molecules. [file 13321_2023_761_MOESM1_ESM.pdf]

A molecule perturbation software library and its application to study the effects of molecular design constraints: Additional file 1

Alan Kerstjens and Hans De Winter

Laboratory of Medicinal Chemistry, Department of Pharmaceutical Sciences, University of Antwerp

Universiteitslaan 1, 2610 Wilrijk, Belgium

Table S1. Number of successfully completed GuacaMol benchmark replicas. We submitted 50 replicas for each of the 20 benchmarks within the benchmark suite. Missing replicas are due to them exceeding the allocated computational time limit.

**Constraint type**	**Number of completed replicas**
None	1000
Local atom	1000
Valence	1000
Local bond	1000
RA atom	1000
Local environment (r = 1)	1000
RA bond	1000
RA environment (r = 1)	999
Local environment (r = 2)	959
RA environment (r = 2)	942

Table S2. Statistical analysis of random molecules’ SAScore differences between the no constraints control group and other groups. Pairwise comparisons were preceded by one-way ANOVA (statistic = 2161.557, p-value < 0.001).

**Comparison**	**Mean SAScore difference**	**Dunnett statistic**	**p-value**
None - Local atom	0.394	-13.472	< 0.001
None - Valence	0.586	-20.052	< 0.001
None - Local bond	0.604	-20.662	< 0.001
None - RA atom	0.761	-26.055	< 0.001
None - Local environment (r = 1)	0.740	-25.327	< 0.001
None - RA bond	1.148	-39.304	< 0.001
None - RA environment (r = 1)	1.645	-56.304	< 0.001
None - Local environment (r = 2)	1.845	-63.164	< 0.001
None - RA environment (r = 2)	3.332	-114.063	< 0.001

Table S3. Statistical analysis of random molecules' QED differences between the no constraints control group and other groups. Pairwise comparisons were preceded by one-way ANOVA (statistic = 356.599, p-value < 0.001).

**Comparison**	**Mean QED difference**	**Dunnett statistic**	**p-value**
None - Local atom	-0.105	16.469	< 0.001
None - Valence	-0.122	19.148	< 0.001
None - Local bond	-0.141	22.118	< 0.001
None - RA atom	-0.139	21.890	< 0.001
None - Local environment (r = 1)	-0.233	36.710	< 0.001
None - RA bond	-0.150	23.603	< 0.001
None - RA environment (r = 1)	-0.191	30.011	< 0.001
None - Local environment (r = 2)	-0.277	43.660	< 0.001
None - RA environment (r = 2)	-0.024	3.825	0.001

Table S4. Average values for QED components of randomly designed molecules using different types of constraints. Note the decay in the number of structural alerts (ALERTS) as constraint stringency increases and the sudden spike in the number of rotatable bonds (ROTB) for RA environment (r = 2) constraints.

Constraint	MW	ALOGP	HBD	HBA	PSA	ROTB	AROM	ALERTS
None	417.16	3.21	2.16	2.78	68.13	5.95	0.005	5.25
Local atom	408.68	3.22	2.04	3.62	70.10	6.45	0.008	3.61
Valence	409.18	3.30	2.27	4.85	68.04	7.26	0.016	3.18
Local bond	408.41	3.23	2.14	4.98	69.21	7.02	0.025	3.02
RA atom	410.06	3.35	2.26	4.95	70.84	7.24	0.018	2.97
Local environment (r = 1)	406.20	3.82	2.02	4.53	64.24	5.42	0.011	2.18
RA bond	408.34	3.57	2.20	4.84	69.00	7.35	0.024	2.80
RA environment (r = 1)	405.54	3.90	2.04	4.51	67.14	7.62	0.028	2.35
Local environment (r = 2)	378.15	3.67	1.50	4.03	58.51	2.91	0.001	1.45
RA environment (r = 2)	382.21	4.45	1.77	3.87	62.41	**14.31**	0.0	2.58

Table S5. Statistical analysis of molecule fitness differences between the no constraints control group and other groups. Pairwise comparisons were preceded by a Kruskal-Wallis test (statistic = 951.677, p-value < 0.001).

**Comparison**	**Median molecule fitness difference**	**Mann-Whitney U-statistic**	**p-value**
None - Local atom	-0.058	472579.5	0.033
None - Valence	-0.020	489620.5	0.421
None - Local bond	-0.073	453589.5	< 0.001
None - RA atom	-0.085	434893.0	< 0.001
None - Local environment (r = 1)	-0.096	436572.0	< 0.001
None - RA bond	-0.128	411981.0	< 0.001
None - RA environment (r = 1)	-0.015	478862.5	0.109
None - Local environment (r = 2)	0.223	613857.0	< 0.001
None - RA environment (r = 2)	0.273	687198.0	< 0.001

Table S6. Statistical analysis of optimized molecules’ SAScore differences between the no constraints control group and other groups. Pairwise comparisons were preceded by one-way ANOVA (statistic = 153.643, p-value < 0.001).

**Comparison**	**Mean SAScore difference**	**Dunnett statistic**	**p-value**
None - Local atom	-0.035	0.618	0.995
None - Valence	-0.075	1.311	0.715
None - Local bond	0.145	-2.538	0.074
None - RA atom	0.203	-3.554	0.003
None - Local environment (r = 1)	0.271	-4.740	< 0.001
None - RA bond	0.612	-10.729	< 0.001
None - RA environment (r = 1)	0.839	-14.704	< 0.001
None - Local environment (r = 2)	0.970	-16.810	< 0.001
None - RA environment (r = 2)	1.438	-24.821	< 0.001

Table S7. Statistical analysis of optimized molecules' QED differences between the no constraints control group and other groups. Pairwise comparisons were preceded by one-way ANOVA (statistic = 10.134, p-value < 0.001).

**Comparison**	**Mean QED difference**	**Dunnett statistic**	**p-value**
None - Local atom	0.001	-0.120	> 0.999
None - Valence	-0.003	0.258	> 0.999
None - Local bond	-0.019	1.834	0.341
None - RA atom	-0.012	1.188	0.803
None - Local environment (r = 1)	-0.047	4.606	< 0.001
None - RA bond	-0.027	2.631	0.058
None - RA environment (r = 1)	-0.035	3.370	0.006
None - Local environment (r = 2)	-0.073	7.01	< 0.001
None - RA environment (r = 2)	-0.017	1.670	0.447

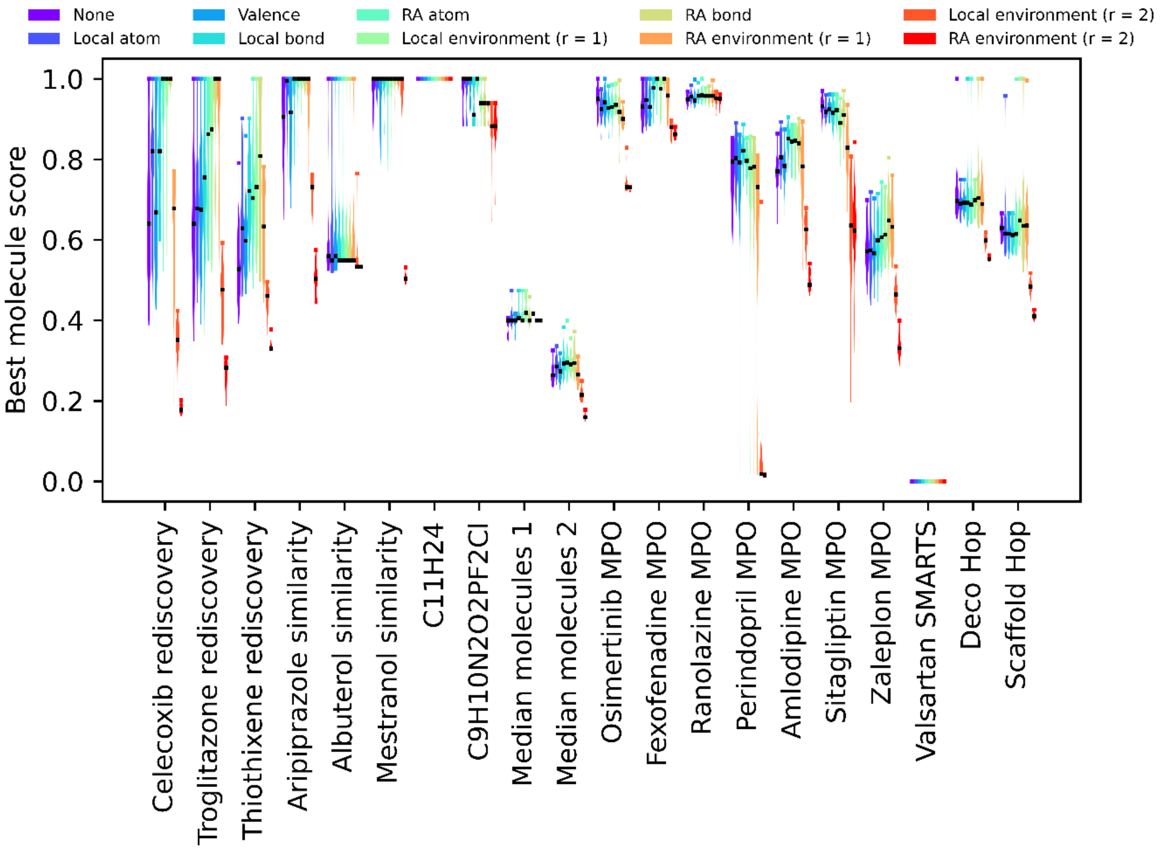

Figure S1. Distributions of top molecule scores, as assessed by the GuacaMol goal-directed scoring functions. Only the best molecule of each population is included. Black squares and colored squares represent median and maximum scores respectively.

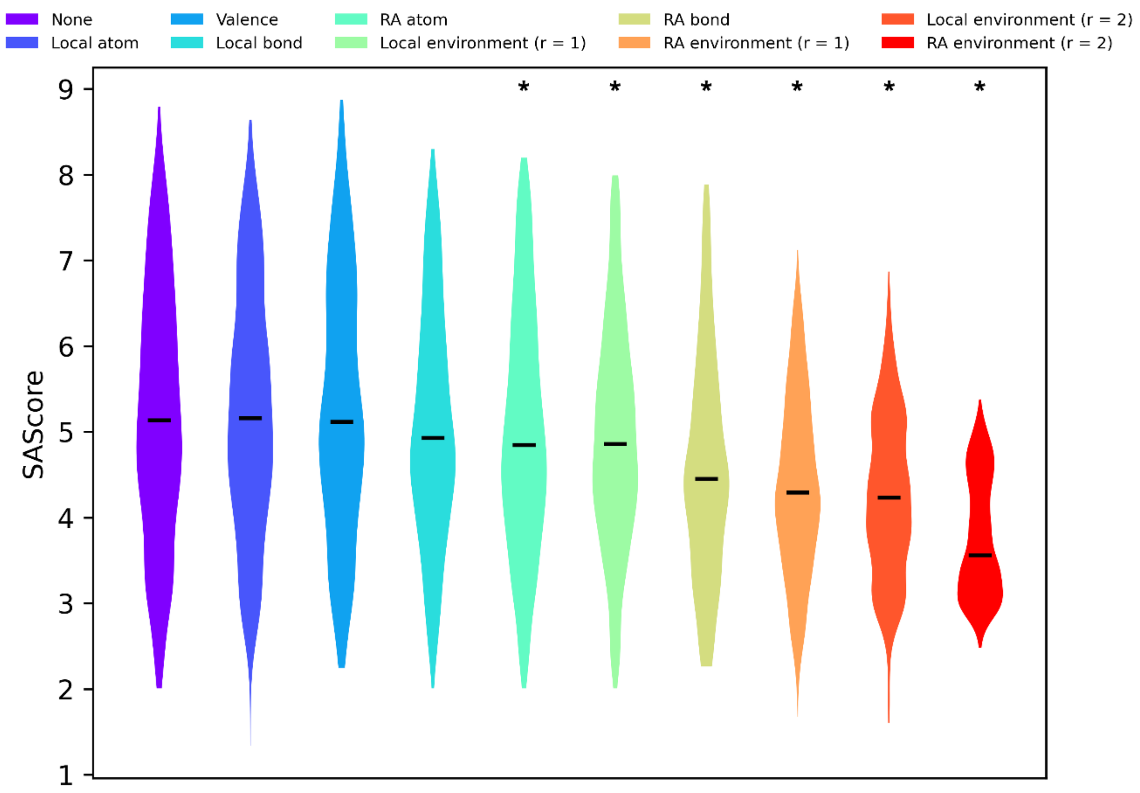

Figure S2. SAScore distributions of molecules designed during the GuacaMol benchmark using different types of constraints. Medians are shown as black lines. Lower SAScores are indicative of an easier synthesis.

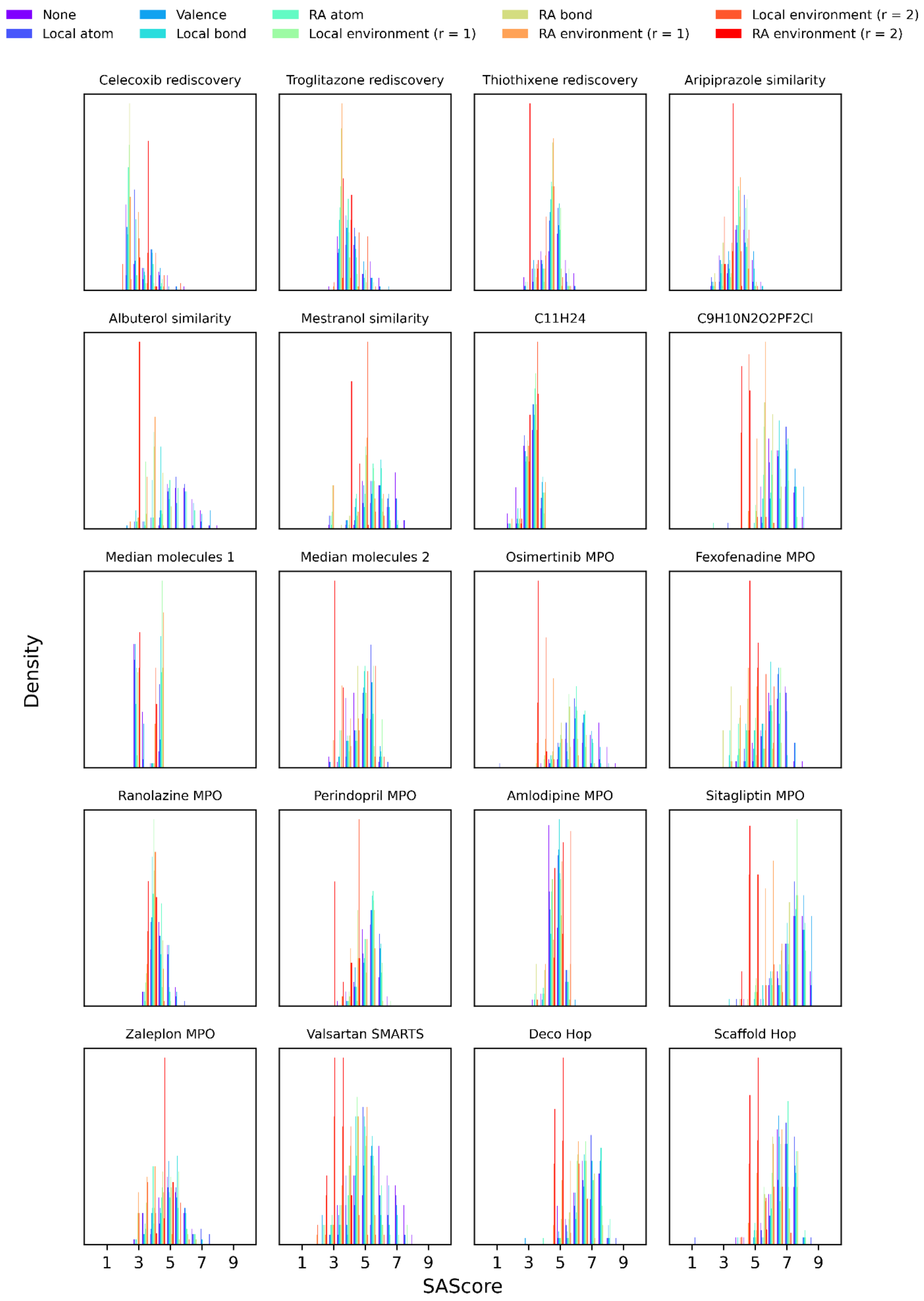

Figure S3. SAScore distributions of molecules designed during the GuacaMol benchmark using different types of constraints broken down by benchmark. Lower SAScores are indicative of an easier synthesis.

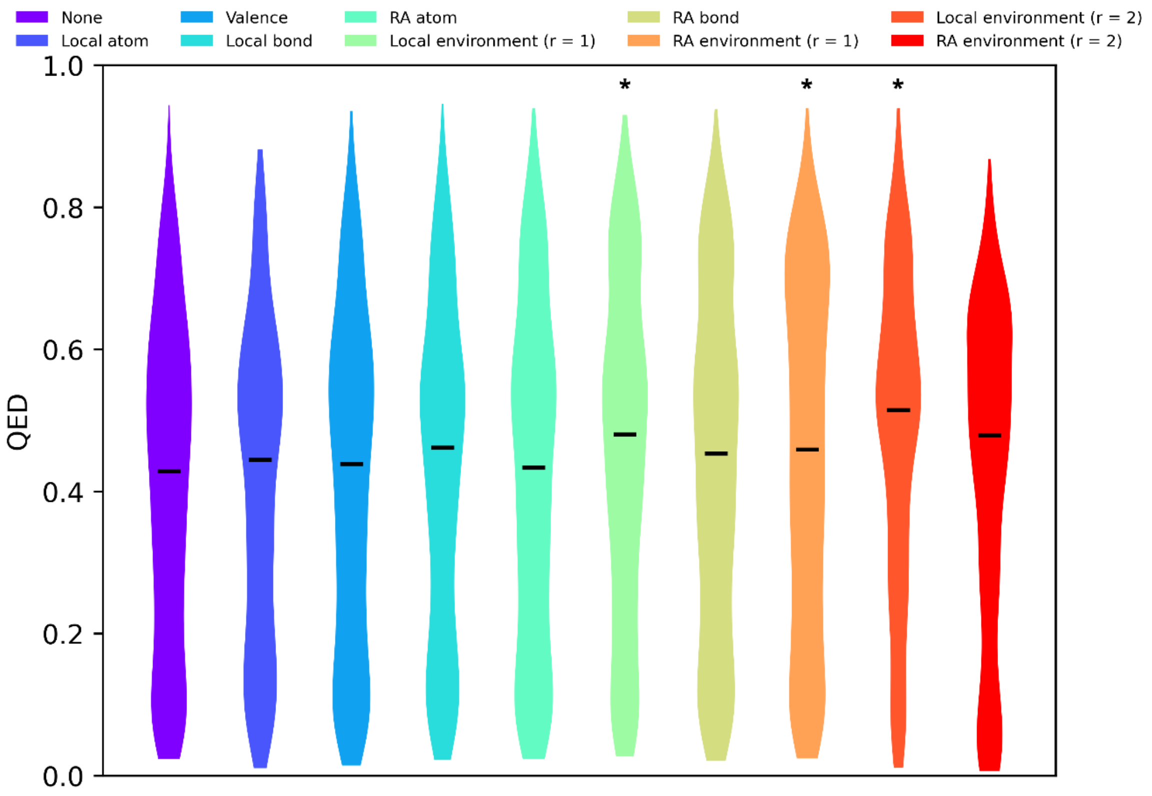

Figure S4. QED distributions of molecules designed during the GuacaMol benchmark using different types of constraints. Medians are shown as black lines. Higher values are indicative of more drug-like molecules.

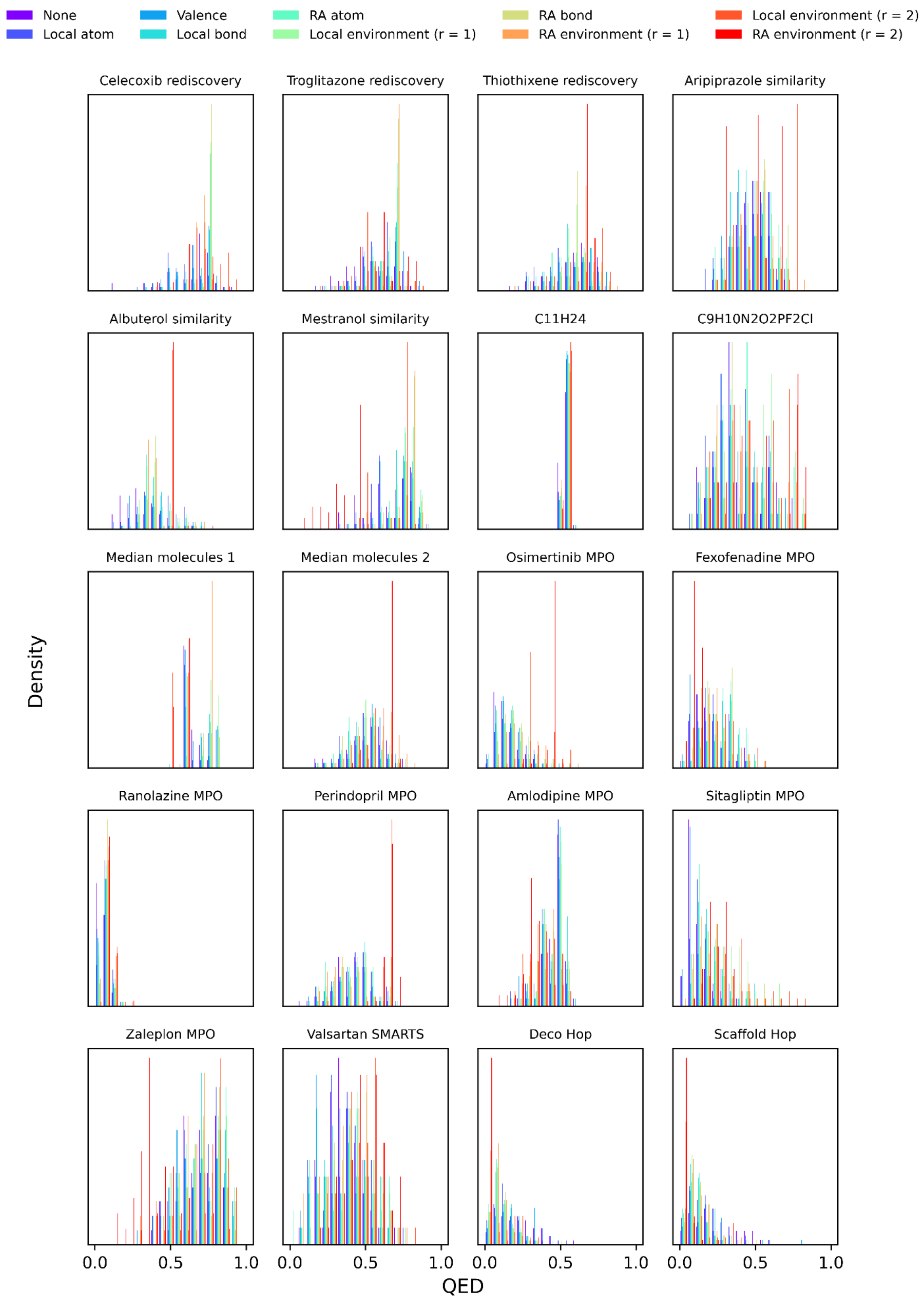

Figure S5. QED distributions of molecules designed during the GuacaMol benchmark using different types of constraints broken down by benchmark. Higher values are indicative of more drug-like molecules.

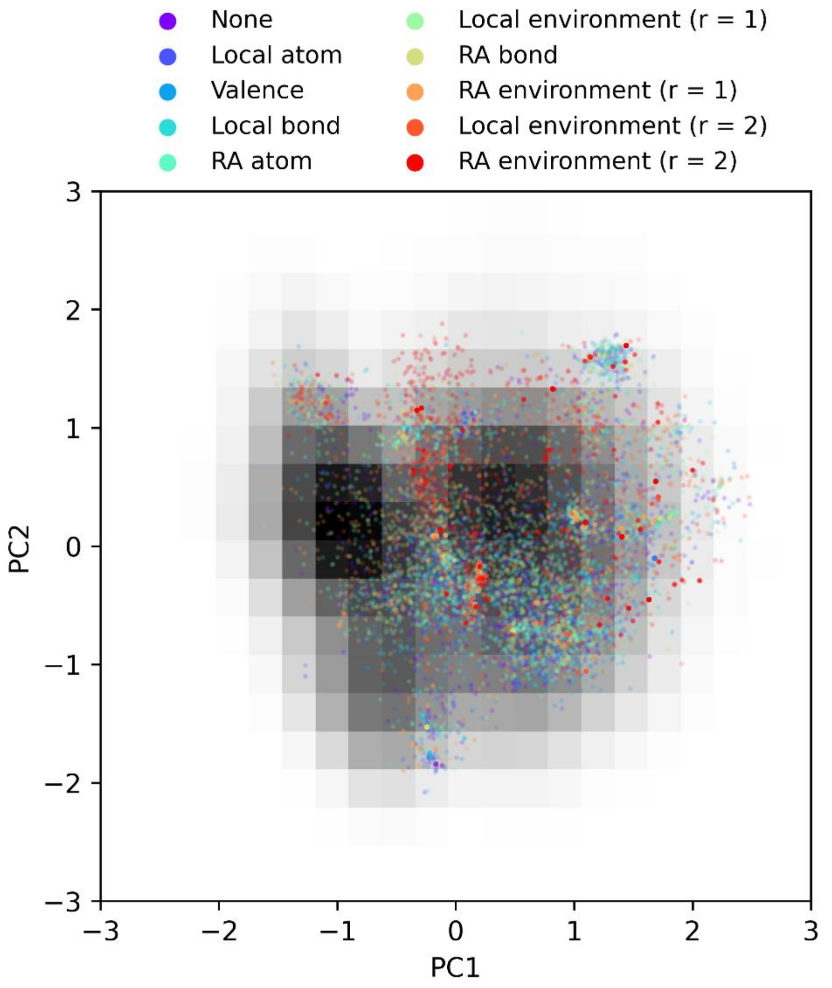

Figure S6. Positions of molecules designed during the GuacaMol benchmark in 2D PCA space. The grayscale grid represents the density of ChEMBL molecules in chemical space on a linear scale, with darker cells being more densely populated.

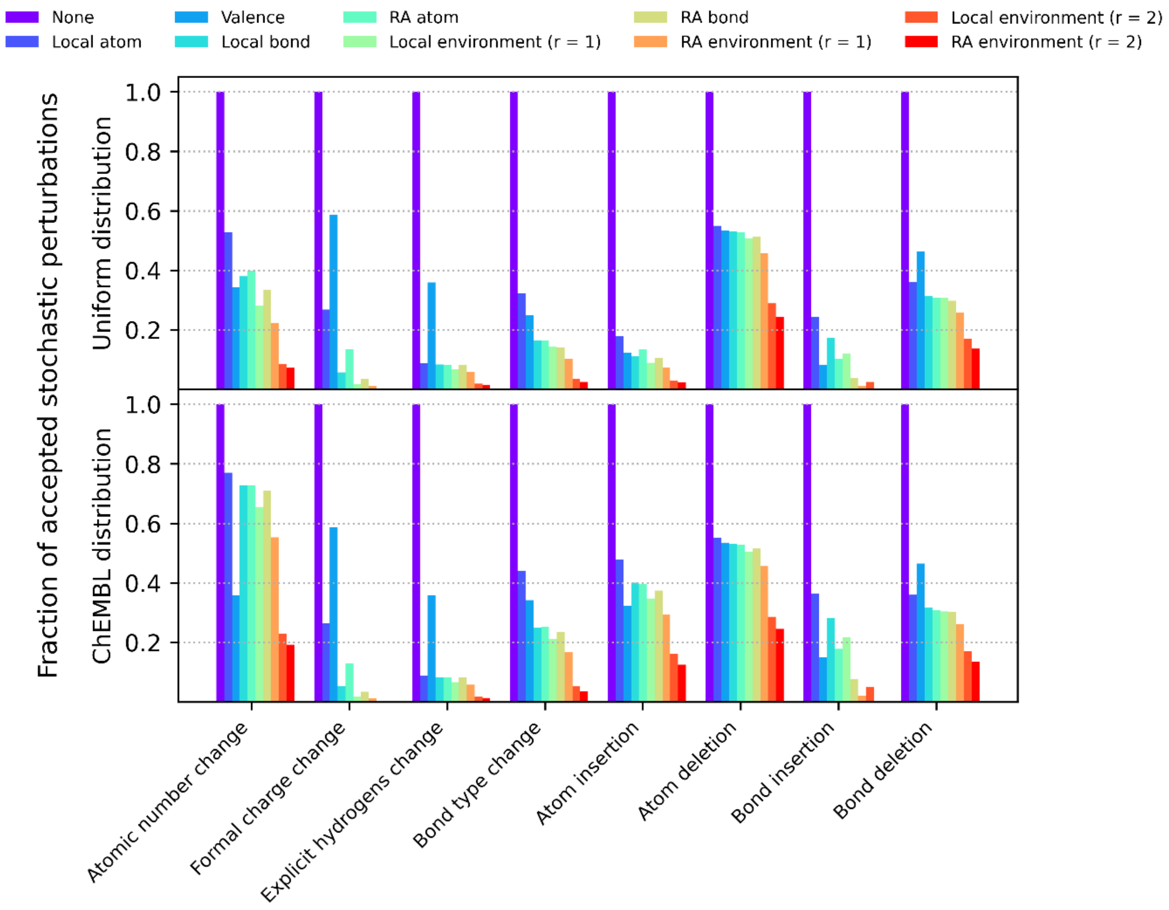

Figure S7. Fraction of stochastically generated perturbations accepted by the molecular constraints, broken down by perturbation type and property value sampling strategy. The upper panel corresponds to uniform random sampling of property values, whereas the lower panel corresponds to weighted random sampling of property values Note that the only perturbation types where property values are sampled are property perturbations (i.e. atomic number, formal charge, explicit hydrogens and bond type changes) and atom/bond insertions.
